# Early detection of dementia with default-mode network effective connectivity

**DOI:** 10.1038/s44220-024-00259-5

**Published:** 2024-06-06

**Authors:** Sam Ereira, Sheena Waters, Adeel Razi, Charles R. Marshall

**Affiliations:** 1 Centre for Preventive Neurology, Queen Mary University of London, London, UK; 2 Max Planck UCL Centre for Computational Psychiatry and Ageing Research, University College London, London, UK; 3 Turner Institute for Brain and Mental Health, School of Psychological Sciences, Monash University, Clayton, Victoria, Australia; 4 Wellcome Centre for Human Neuroimaging, Institute of Neurology, University College London, London, UK; 5 CIFAR Azrieli Global Scholars Program, CIFAR, Toronto, Ontario, Canada; 6 Department of Neurology, Royal London Hospital, London, UK

## Abstract

Altered functional connectivity precedes structural brain changes and symptoms in dementia. Alzheimer’s disease is the largest contributor to dementia at the population level, and disrupts functional connectivity in the brain’s default-mode network (DMN). We investigated whether a neurobiological model of DMN effective connectivity could predict a future dementia diagnosis at the single-participant level. We applied spectral dynamic causal modeling to resting-state functional magnetic resonance imaging data in a nested case–control group from the UK Biobank, including 81 undiagnosed individuals who developed dementia up to nine years after imaging, and 1,030 matched controls. Dysconnectivity predicted both future dementia incidence (AUC = 0.82) and time to diagnosis (*R* = 0.53), outperforming models based on brain structure and functional connectivity. We also evaluated associations between DMN dysconnectivity and major risk factors for dementia, revealing strong relationships with polygenic risk for Alzheimer’s disease and social isolation. Neurobiological models of effective connectivity may facilitate early detection of dementia at population level, supporting rational deployment of targeted dementia-prevention strategies.

There is currently intense interest in identifying strategies to reduce the growing population burden of dementia^1^. Clinical syndromes of dementia are caused by multiple neuropathologies that typically co-occur within individuals^2^. Alzheimer’s disease (AD) pathology is the most important contributor to dementia at the population level, and is associated with distinct patterns of pathological protein deposition and altered neural function that precede the development of structural brain changes and clinical symptoms by a period of years^3^. The ability to reliably detect early changes in neural function associated with AD would provide a platform for the development of individualized dementia-prevention strategies.

Resting-state functional magnetic resonance imaging (rs-fMRI) is increasingly used as a tool for characterizing connectomic bio-markers in AD^4^. It measures endogenous fluctuations in blood-oxygen-level-dependent (BOLD) signal across the brain—which in turn reflect regional neural activation—while a participant lies in an MRI scanner at rest. A map of functional connectivity can be estimated by computing correlations between BOLD time-series from different brain regions^5^. When rs-fMRI is applied to people with AD, or its precursor, mild cognitive impairment (MCI), there are substantial changes in functional connectivity at the group level, when contrasted with healthy controls^6–9^. Similar changes have been identified in individuals who do not yet have MCI or AD but are considered high risk due to genetic polymorphisms^10–12^, mutations for autosomal dominant AD^13^, a family history of AD^14^ or a high burden of pathogenic amyloid and tau proteins^15–20^. Altered functional connectivity—measured with rs-fMRI—is therefore widely considered a potential preclinical bio-marker of AD^21^. However, it has not previously been shown to allow single-participant level identification of future dementia risk in a population-based cohort.

The brain regions most commonly implicated in altered functional connectivity in AD are those within the default-mode network (DMN), which is hypothesized to be selectively vulnerable to AD neuropathology^22^. The DMN is typically described as having a core set of brain regions, which includes the medial prefrontal cortex (mPFC), posterior cingulate cortex or precuneus, and bilateral inferior parietal cortices, as well as a set of supplementary brain regions such as the medial temporal lobes and temporal poles^23^. The DMN was initially described as a network of regions that co-activate during a task-negative state in functional imaging studies^24^. In other words, these brain regions seem to be more active when a participant is at rest. However, research shows that the DMN is implicated in several high-level cognitive processes such as social cognition and mental time-travel^23,25^, resulting in a contemporary view that the DMN furnishes an individual with their narrative sense of self^26^.

Although findings of altered functional connectivity in the DMN have led to claims that dementia is a syndrome of dysconnectivity, the exact connectivity changes observed are inconsistent across studies^4,27^, and are occasionally undetectable^28^. This is perhaps due to methodological limitations associated with defining connectivity on the basis of time-series correlations that overlook biophysical constraints and the established neurobiology of neural circuit function. An alternative approach in connectomics is to fit a neurobiologically informed circuit model to the functional neuroimaging data to characterize the excitatory and inhibitory connections between different brain regions, that is, effective connectivity^29^. Moving beyond correlations in brain activity, effective connectivity describes the causal influence of one brain region over another, by modeling the underlying neural signals that generated the observed data.

Correlations in BOLD activity among brain regions in a network can be explained by an enormous number of possible underlying neural circuitries. With dynamic causal modeling (DCM), multiple putative circuit models of effective connectivity can be compared with each other using model comparison procedures, and the best explanation for the observed data is identified. Thus, effective connectivity provides a more nuanced description of neural connectivity and is likely to detect features that would otherwise be missed when mapping functional connectivity derived from observed BOLD signal. These connectomic subtleties, effective connectivity parameters, are likely to afford discriminative and predictive clinical value, for individualized precision medicine, over and above the correlations measured in functional connectivity^30^.

In neurodegeneration and ageing, neural connections and neurovascular coupling are both impacted^31^, and here DCM becomes particularly useful. As functional connectivity is a multiplexed signal of neural, haemodynamics and noise components, any observed changes in functional connectivity do not differentiate between changes in neural circuitry, haemodynamics or both. DCM, on the other hand, models the neural, haemodynamics and noise components of BOLD separately. Effective connectivity mapping with DCM has been used to successfully discriminate between people with semantic dementia and healthy controls^32^, and also to predict which people with Parkinson’s disease are likely to experience hallucinations^33^. A small number of studies have estimated effective connectivity differences between people with AD or MCI, and healthy controls^34–39^, and have also detected differences in small samples of undiagnosed individuals at high-risk for AD^40,41^. However, to the best of our knowledge, there has been no work on the potential for effective connectivity to make predictions about dementia incidence at single-participant level.

Here we investigated whether effective connectivity changes in the DMN can be used to make early predictions about dementia incidence and prognosis in a population cohort. To that end we constructed a nested case-control study using the UK Biobank (UKB) cohort, among which a sample have developed incident dementia in the years since neuroimaging data acquisition. To ensure that the analysis was ecological and reflected the range of dementia pathologies within the population, we used all-cause dementia outcomes rather than restricting the analysis to those with Alzheimer’s disease. We analyzed rs-fMRI data from individuals who developed dementia and a large sample of matched controls. We applied spectral DCM^30^—a technique that fits generative neural and haemodynamic models to the cross-spectra of BOLD time-series from rs-fMRI data—to estimate effective connectivity. We predicted that there would be detectable differences in DMN effective connectivity years before people were diagnosed with dementia, and that these differences would be large enough to make meaningful out-of-sample predictions about future dementia incidence. We also predicted that these early patterns of dysconnectivity would be associated with exposure to known risk factors, particularly polygenic risk for AD as the key driver of AD pathological change, and social isolation due to the role of the DMN in social cognition^23^.

## Results

**Fig. 1:**
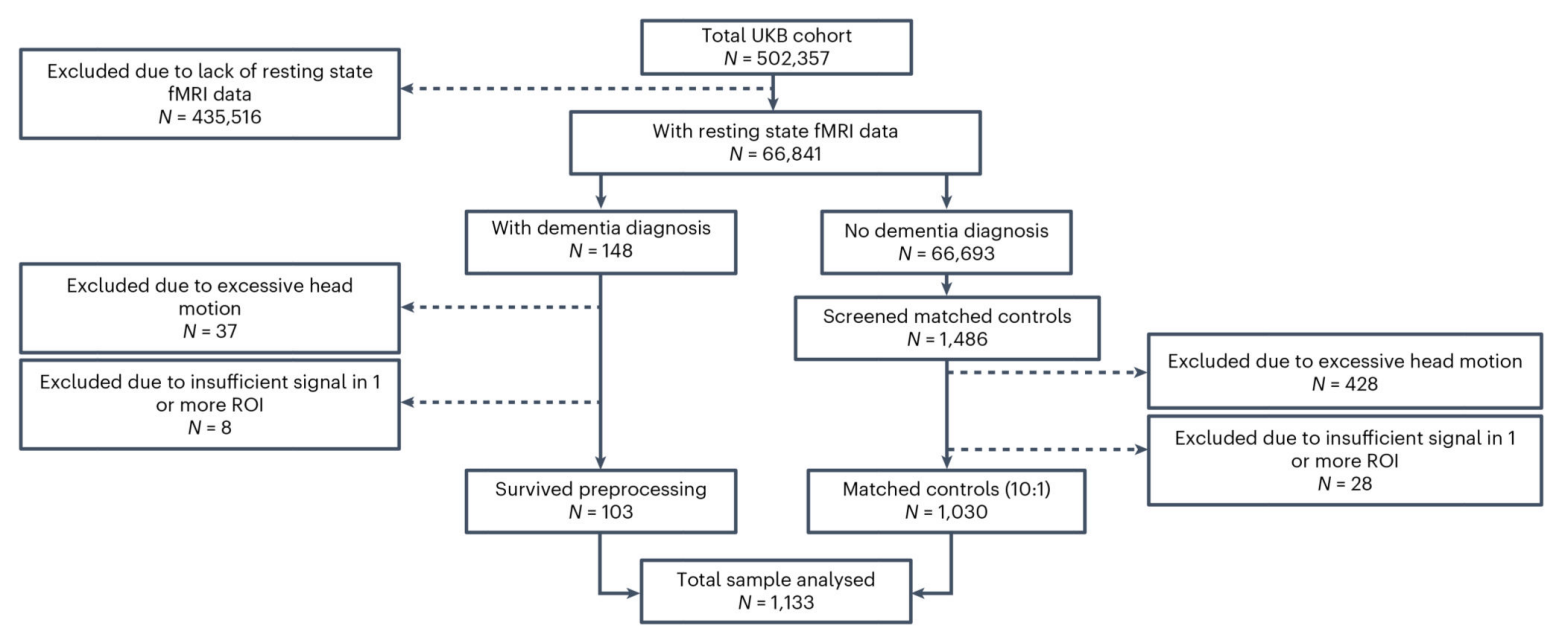
Recruitment flowchart. Dementia cases and matched controls were selected from the UKB cohort. Once the final usable sample of dementia cases was determined (*N* = 103), matched controls were iteratively selected and screened to ensure they satisfied fMRI preprocessing criteria, until there were ten suitable matched controls for each individual case (*N* = 1,030).

After exclusions for image quality and excessive in-scanner head motion (see Methods), our final usable sample included 103 dementia cases (22 individuals with prevalent dementia and 81 who later developed incident dementia) and 1,030 matched controls (see Fig. 1). The 81 incident cases had a median time to diagnosis of 3.7 years (range = 0.4–8.5). The total sample had a mean age of 70.4 at the time of MRI data acquisition. Cases and controls were matched on age, sex, ethnicity, handedness and geographical location of the testing center (see Extended Data Table 1 for sample characteristics). Cases performed worse than controls in four cognitive tests, which were analyzed as part of this study (see Methods and Supplementary Table 3). Although most of our case-sample were prediagnostic, their lower cognitive test scores might reflect objective evidence of cognitive decline. Alternatively, these results might reflect a reduced cognitive reserve in this sample.

**Fig. 2:**
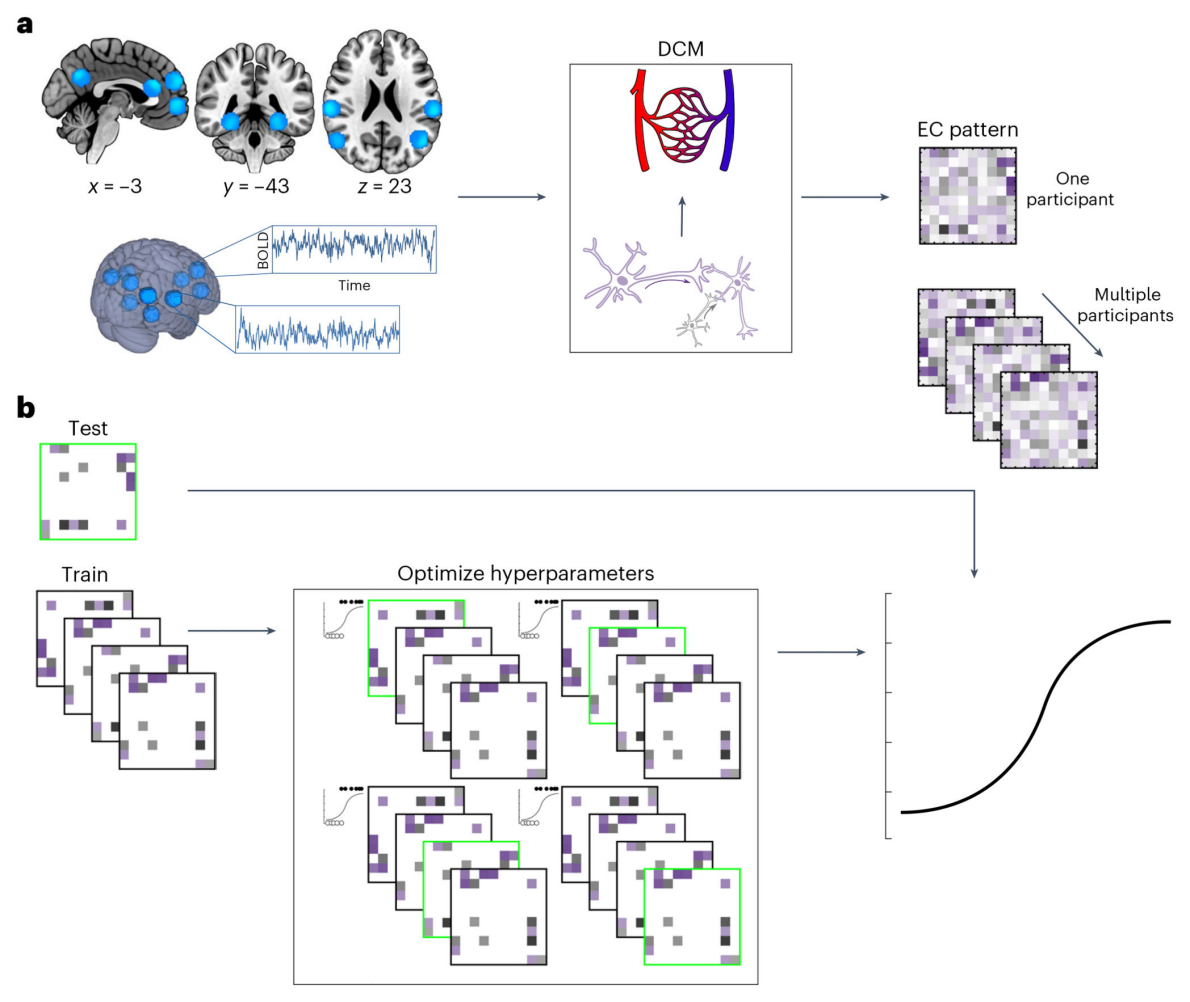
Graphical summary of the analysis pipeline. **a**, Ten ROIs were used to define the default-mode network. There are four mid-line ROIs shown in the sagittal section (top left), two medial temporal ROIs shown in the coronal section (top middle), and four lateral temporal and parietal ROIs shown in the axial section (top right). For each participant, voxels were only selected within the sphere if supra-threshold activation was detected. Lighter shades of blue indicate voxels that were selected more frequently across participants. BOLD time-series were extracted from each of the ten ROIs. A spectral DCM was fitted to these BOLD time-series data. The DCM optimizes effective connectivity parameters to find the best explanation for the observed BOLD time-series in terms of excitatory (purple) and inhibitory (gray) neural connections, as well as altered blood flow that would be expected to result from this neural activity. Each participant’s effective connectivity (EC) pattern is estimated separately and is represented as a 10 × 10 EC matrix, where each cell in the matrix shows the magnitude and valence (excitatory or inhibitory) of a connection between a pair of ROIs. **b**, Bayesian model reduction is applied to the EC matrix to eliminate unnecessary parameters and find the most parsimonious model to explain the observed data, at the group level. The resulting sparse EC patterns are used to train regularized logistic regression models to predict dementia incidence using nested cross-validation. A single participant (or random subgroup) is left out of the analysis as a test set, highlighted in green. All remaining participants constitute a training set. The hyperparameters of the regression model are optimized on this training set with new nested test and train sets within the outer training set. Once optimal hyperparameters are selected, the optimized model is trained on the full outer training set and tested on the original left-out sample. This procedure is iterated such that every participant (or subgroup) is used once as a test set.

The analysis pipeline is illustrated in Fig. 2. For each participant, BOLD time-series were extracted from ten pre-defined regions-of-interest (ROIs), which together defined our DMN. The network included four mid-line ROIs: the precuneus (PRC), anterior medial prefrontal cortex (amPFC), dorsomedial prefrontal cortex (dmPFC) and ventro-medial prefrontal cortex (vmPFC); one ROI in each medial temporal lobe, in the left and right parahippocampal formations (lPHF/rPHF); and four lateral ROIs, the right intraparietal cortex (rIPC), left intra-parietal cortex (lIPC), right lateral temporal cortex (rLTC) and left lateral temporal cortex (lLTC). See Methods for further details on these ROIs.

A fully connected DCM was fitted to the cross-spectra of these time-series data (spectral DCM) to estimate the effective connectivity between each and every pair of ROIs in the ten-node network (Fig. 2a).

### Effective connectivity predicts who will get dementia

**Fig. 3:**
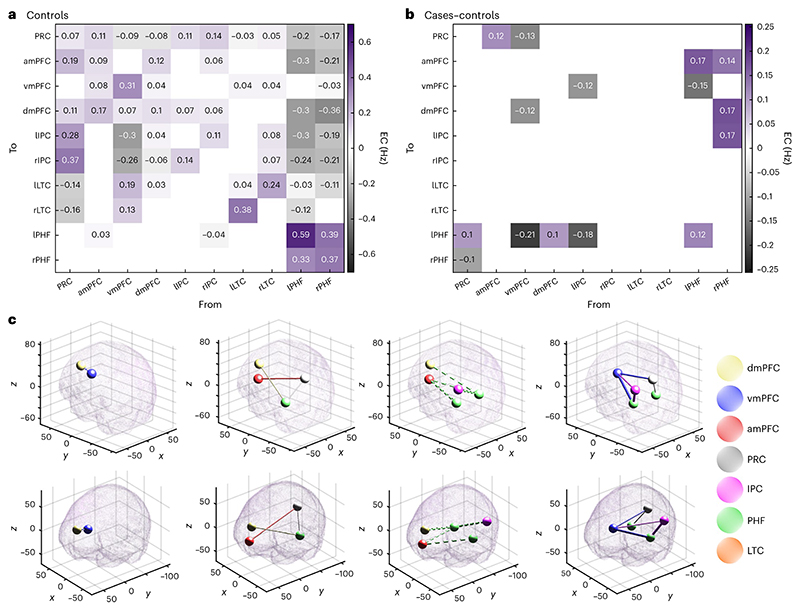
Effective connectivity differences between cases and controls. **a**, Bayesian model average of effective connectivity in healthy controls. Each cell shows the effective connectivity (EC), in hertz, between a pair of regions. Gray and purple colours indicate inhibitory and excitatory connections, respectively. Cells along the diagonal represent auto-inhibitory connections, as unitless scaling parameters. Only parameters with an at least 99% posterior probability of being non-zero (amounting to a very strong evidence) are shown. **b**, Bayesian model average of the difference in effective connectivity between cases and controls. Gray indicates a change towards increased inhibition (reduced excitation), whereas purple indicates a change towards increased excitation (reduced inhibition). Only parameters with an at least 99% posterior probability of being non-zero are shown. **c**, Effective connectivity differences between cases and controls visualized in Montreal Neurological Institute (MNI) space. Each tube represents a connection change. Solid tubes represent connections that are strengthened in cases compared with the controls; dashed tubes represent connections that are attenuated in cases compared with the controls; the thickness of the tube represents the magnitude of the connection change; and the color of the tube represents the brain region from where the connection originates. The top row and bottom rows show the same data but from two different angles. The four columns display the following, respectively: attenuated excitatory connections, strengthened excitatory connections, attenuated inhibitory connections, strengthened inhibitory connections.

Bayesian model reduction and averaging were applied (see Methods) to estimate the simplest effective connectivity map to explain group-level differences between dementia cases and controls (Fig. 3) while controlling for age, sex and in-scanner head motion. There was very strong evidence (posterior probability > 0.99) for 15 connectivity parameters that differed between cases and controls. The three largest connectivity changes seen in the dementia cases were: increased inhibition from the vmPFC to lPHF, increased inhibition from lIPC to the lPHF, and attenuated inhibition from the rPHF to the dmPFC.

**Fig. 4:**
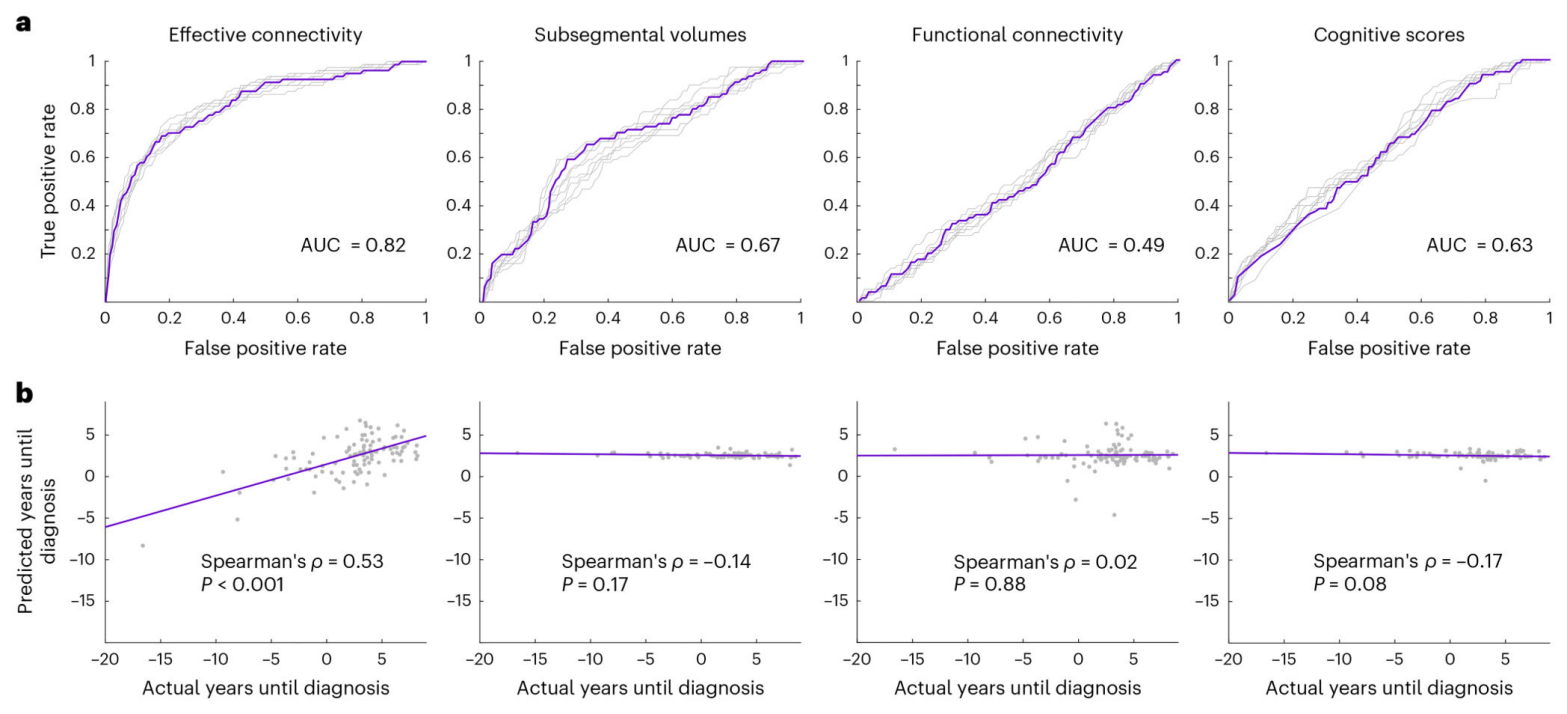
Performance of classification and prognostication models. **a**, ROC curves for regularized logistic regression models trained on effective connectivity parameters, gray matter volumes, functional connectivity or cognitive scores to classify undiagnosed dementia cases from controls. A ROC curve is generated by taking the mean curve across all test-folds of cross-validation. In each plot there are nine ROC curves because nine iterations of stratified *K*-fold cross-validation were performed. The purple curves indicate the iteration that generated the median AUC across iterations. This median AUC is indicated at the bottom right of the plot. **b**, Scatter-plots showing the performances of regularized linear regression models trained on the same data types as in **a**, to predict the time until dementia diagnosis across 102 dementia cases (81 undiagnosed and 21 with a pre-existing diagnosis). All statistical tests are two-sided, and *P* = 2 × 10^−8^ for the effective connectivity prognosticator (left-most scatter-plot).

These 15 connectivity parameters (Fig. 3b) were used to train an elastic-net logistic regression model to predict future dementia diagnosis in stratified *K*-fold cross-validation. The model was trained on the entire dataset, including prevalent cases who were already diagnosed with dementia, but performance was evaluated exclusively on classification between prediagnostic cases and their matched controls. Using receiver-operating characteristic (ROC) analysis, we found the model to have excellent discriminative performance (Fig. 4a) with an area under the curve (AUC) of 0.824 (range = 0.79–0.843). See Methods for further details on how the AUC was computed. As a sensitivity analysis, a classifier was also trained on the full model of 100 effective connectivity parameters. This yielded a marginally reduced AUC of 0.816 (range = 0.807–0.842).

### Effective connectivity predicts time until dementia diagnosis

**Fig. 5:**
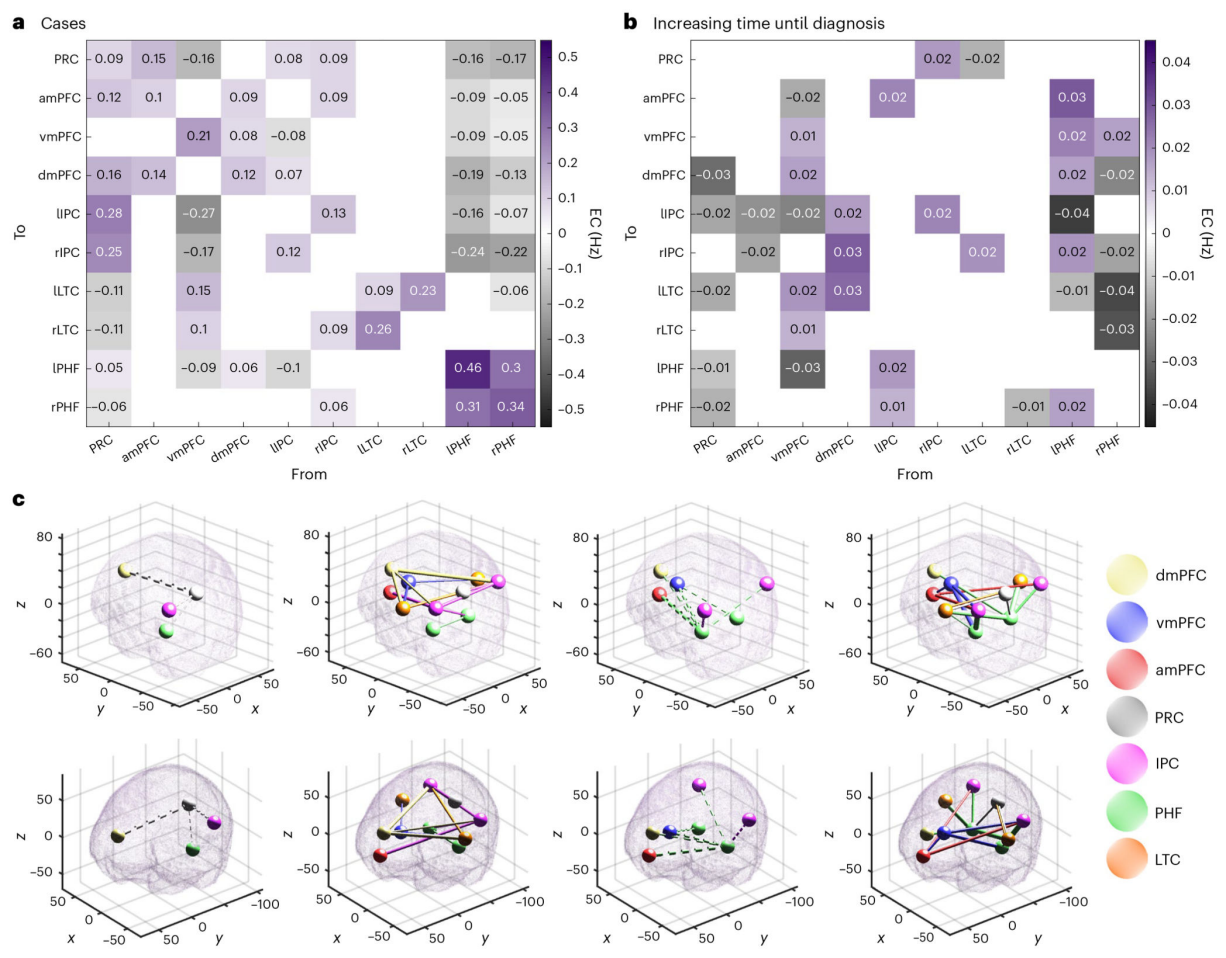
Effective connectivity changes associated with the time until dementia diagnosis. **a**, Bayesian model average of effective connectivity in cases. Each cell shows the effective connectivity (EC), in hertz, between a pair of regions. Gray and purple colours indicate inhibitory and excitatory connections, respectively. Cells along the diagonal represent auto-inhibitory connections, as unitless scaling parameters. Only parameters with an at least 99% posterior probability of being non-zero are shown. **b**, Bayesian model average of the changes in effective connectivity, among cases, associated with a longer time until diagnosis. Gray indicates a change towards increased inhibition (reduced excitation), whereas purple indicates a change towards increased excitation (reduced inhibition). Only parameters with an at least 99% posterior probability of being non-zero are shown. **c**, Effective connectivity changes. Visualization follows the same format as Fig. 2c, but with connectivity changes associated with time until diagnosis, rather than differences between cases and controls.

To assess the potential role of DMN effective connectivity in prognostication, we ran an analysis that only used the case cohort. We used Bayesian model reduction and averaging (see Methods) to estimate the simplest effective connectivity map to explain the inter-individual variation associated with the time until dementia diagnosis while controlling for age, sex and in-scanner head motion. The time until diagnosis was negatively valued for participants who already had a diagnosis of dementia at the time of data acquisition. There was a very strong evidence (posterior probability > 0.99) for 37 connectivity parameters that were associated with the time until diagnosis (Fig. 5), including the three connections, described above, that showed the largest difference between cases and controls (Fig. 3).

These 37 connectivity parameters were used to train an elastic-net regularized linear regression model to predict time until diagnosis in *K*-fold cross-validation. There was a positive correlation between actual time until diagnosis and predicted time until diagnosis (Spearman’s *ρ* = 0.53, *P* = 2 × 10^−8^). As a sensitivity analysis, a linear regression model was also trained on the full model of 100 effective connectivity parameters. This still yielded a positive correlation between the actual and predicted times until diagnosis, but the effect was reduced (Spearman’s *ρ* = 0.36, *P* = 1.9 × 10^−4^).

### Comparisons with alternative metrics

To see how effective connectivity compares as a diagnostic and prognostic tool with other MRI-based markers, we trained predictive models using exactly the same methods as above, but using volumetric data and functional connectivity data instead of effective connectivity parameters.

We used 40 hippocampal and parahippocampal subsegmental volumetric features for the volumetric-based models (see Methods). Interestingly, we found that dementia-related change in effective connectivity was negatively associated (*R*^2^ = 0.012, *P* = 0.0002) with the mean volume across these subsegmental regions (see Methods). In an exploratory post-hoc analysis, we found that the three subsegmental volumes with the strongest negative association with effective connectivity change were: the left head of hippocampal CA3 (*R*^2^ = 0.014, *P* = 6.73 × 10^−5^), left body of subiculum (*R*^2^ = 0.013, *P* = 1.03 × 10^−4^) and left anterior parahippocampal gyrus (*R*^2^ = 0.012, *P* = 2.12 × 10^−4^). These findings suggest that the effective connectivity changes that we observed probably reflect an AD-like pathology in which the earliest volume loss is in the medial temporal lobes, particularly the entorhinal cortex. Despite the fact that effective connectivity change and volume loss may reflect the same pathological process, the elastic-net logistic regression classifier trained on volumetric data (Fig. 4a) yielded only moderate diagnostic value with an AUC of 0.671 (range = 0.62–0.69). The elastic-net linear regression prognosticator (Fig. 4b) performed at chance level (Spearman’s *ρ* = −0.14, *P* = 0.17).

For the functional connectivity analysis, we computed Fisher *z*-transformed Pearson coefficients between every pair of ROIs in the same network that we used for the effective connectivity analysis. This yielded 45 functional connectivity values (see Methods). The elastic-net logistic regression classifier (Fig. 4a) performed at chance level with an AUC of 0.491 (range = 0.478–0.517); the elastic-net linear regression prognosticator (Fig. 4b) also performed at chance level (Spearman’s *ρ* = 0.02, *P* = 0.88).

Given the considerable differences in cognitive task data between cases and controls, we also tested whether cognitive data alone could predict both a future dementia diagnosis and the time until diagnosis. The elastic-net logistic regression classifier (Fig. 4a) yielded moderate performance with an AUC of 0.628 (range = 0.606–0.641). The elastic-net linear regression prognosticator (Fig. 4b) performed at chance level (Spearman’s *ρ* = −0.17, *P* = 0.08).

Comparing all four data types, effective connectivity parameters yielded the best classification performance for predicting future dementia diagnosis, and was the only data-type that yielded better-than-chance prognostication (prediction of time until dementia diagnosis).

### Associations between risk factors and effective connectivity

Finally, we conducted an exploratory analysis to investigate whether the effective connectivity changes might represent the effects of major risk factors for dementia. We first defined an effective connectivity index for each participant, which was simply the probability of dementia, outputted by a case-control classifier trained on effective connectivity parameters with leave-one-out cross-validation (see Methods). This value summarizes the extent to which an individual’s DMN effective connectivity pattern conforms to a dementia-like phenotype rather than a control-like phenotype, where a value of 1 indicates a dementia-like pattern and a value of 0 indicates a control-like pattern.

**Fig. 6:**
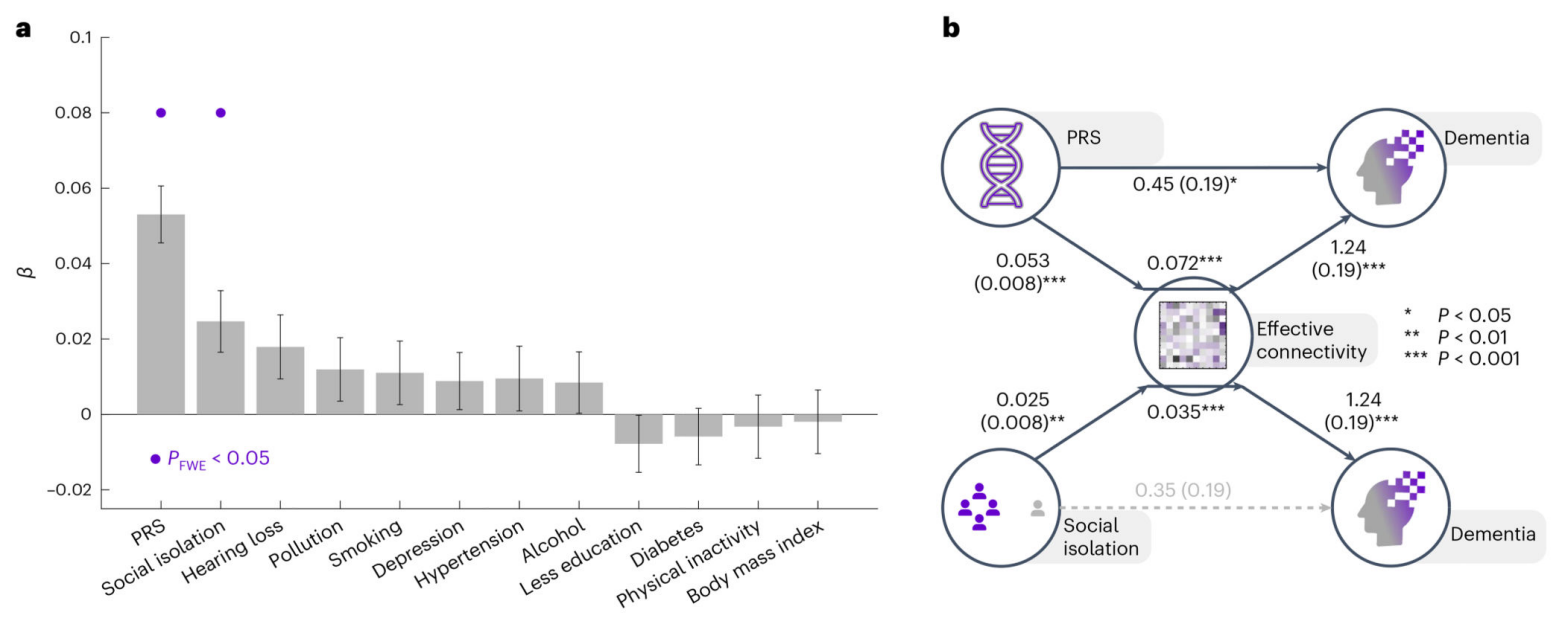
Associations between DMN dysconnectivity and risk factors. **a**, Standardized regression coefficients (*β*) from 12 different linear regression models, testing for associations between PRS and 11 modifiable risk factors for dementia and DMN EC index. Each bar reflects the estimated coefficient from a regression model across all *N* = 1,133 participants, where age, sex and social deprivation (Townsend) score were included as covariates of no-interest. Error bars show standard error of the regression coefficients. Any regression coefficients that are statistically significant after correcting for multiple comparisons are labeled with a purple dot; *P*_FWE-corrected_ = 4.4 × 10^−11^ for PRS, *P*_FWE-corrected_ = 0.028 for social isolation. All statistical tests are two-sided. See Supplementary Table 1 for more detailed results. **b**, Results of two mediation models indicating that there are significant indirect paths whereby EC index mediates the association between social isolation and dementia and partially mediates the association between polygenic risk score and dementia. Each path is labeled with regression coefficient and standard error in brackets. All statistical tests are two-sided.

For each individual, we then extracted data from UKB describing the modifiable risk factors identified in the 2020 Lancet commission on dementia^1^ (see Methods), as well as each participant’s AD polygenic risk score (PRS). For each risk factor, we ran a separate weighted linear regression model, across the entire cohort of cases and controls (*N* = 1,133), to measure the association between effective connectivity index and that specific risk factor, controlling for age, sex and social deprivation (Townsend) score (Fig. 6a and Supplementary Table 1). After correcting for multiple comparisons, the AD PRS was strongly associated with effective connectivity index (*β* = 0.053, *P* = 3.7 × 10^−12^, *P*_FWE-corrected_ = 4.4 × 10^−11^), and this association was much stronger than any association between effective connectivity index and a modifiable risk factor (Fig. 6a). This corroborates the earlier finding of a negative association between hippocampal and parahippocampal volume and effective connectivity change, and suggests that these EC changes probably represent Alzheimer’s pathology rather than a more general reflection of brain health.

We constructed a mediation model (Fig. 6b) to see whether effective connectivity index mediated any of the relationship between PRS and dementia incidence. By including the effective connectivity index as a mediator, the direct path coefficient from PRS to dementia incidence was reduced from 0.5 (*P* = 0.0007) to 0.45 (*P* = 0.017). There was a significant indirect mediated path (*β* = 0.07, *P* < 0.001), which explained away 10% of the association between PRS and dementia incidence. These results indicate that DMN effective connectivity partially mediates the role of genetic risk in dementia pathogenesis.

For the modifiable risk factors, social isolation was the only variable that showed a significant association with effective connectivity index (*β* = 0.025, *P* = 0.003, *P*_FWE-corrected_ = 0.028). This association demonstrated that individuals with more self-reported social isolation were more likely to have a ‘dementia-like’ pattern of DMN effective connectivity. To see whether social isolation was simply an early sign of cognitive impairment, we constructed a composite score of cognitive ability (see Methods) and tested whether this was correlated with social isolation. These variables were not correlated (Spearman’s *ρ* = 0.05, *P* = 0.11), consistent with social isolation being a cause rather than a consequence of the dementia process.

We constructed a mediation model (Fig. 6b) to test whether effective connectivity index might mediate the known association between social isolation and dementia incidence. After accommodating for a hypothesized mediating effect of effective connectivity index, we detected a significant indirect path from social isolation to dementia, mediated by effective connectivity index (*P* < 0.001). Furthermore, an association between social isolation and dementia incidence (*P* = 0.037) was rendered non-significant (*P* = 0.07), after accounting for this mediator. To further test the biological plausibility of this model, we repeated this mediation analysis, excluding the 22 prevalent cases and their 220 matched controls. This yielded comparable results, with a significant indirect mediation path (*P* < 0.001), and a direct path from social isolation to dementia (*P* = 0.043) that was rendered non-significant by including the mediator (*P* = 0.09). These results show that prediagnostic effective connectivity changes, in the DMN, mediate an association between premorbid social isolation and subsequent dementia incidence. Taken together, prediagnostic DMN dysconnectivity appears to be a consequence of both genetic and environmental risk factors.

## Discussion

Our findings show that a neurobiologically informed model of DMN effective connectivity can enable accurate predictions about whether and when an individual will develop dementia. The performance of our effective connectivity-based classifier exceeded that of classifiers based on volumetric and functional connectivity data, both in our analyses, and also when comparing it with past works using structural MRI data as a unimodal predictor of future conversion to dementia^42^.

From a clinical perspective, this suggests that rs-fMRI could become a tool for identifying a neural network signature of dementia risk early in the pathological course of the disease. This type of non-invasive early detection of dementia is an increasingly valuable goal, particularly with the arrival of disease-modifying drugs. Recent clinical trials have shown promise for amyloid-β-targeting monoclonal antibodies, which are modifying the disease trajectory in AD for the first time^43,44^, supposedly with greater therapeutic potential when started earlier in the disease process. Early detection of dementia risk is also important in the context of targeted risk reduction strategies irrespective of underlying pathology^1^. Whereas pathology-specific biomarkers can guide disease-modifying molecular therapies, non-specific biomarkers for all-cause dementia, such as those developed in this study, will be useful for identifying who is most likely to benefit from lifestyle changes and public health interventions, and when these interventions are likely to have the biggest impact.

Recent research on early detection of dementia tended to prioritize biomarkers that directly reflect pathogenic protein deposition in AD, such as cerebrospinal fluid analysis for amyloid beta and tau proteins. However, these markers have limited predictive ability among healthy population cohorts because a majority of individuals remain asymptomatic during follow up (for example, >90% of those with amyloid beta positivity remain asymptomatic over five years^45^). Plasma levels of phosphorylated tau are highly predictive of AD neuropathology^46^ and can also accurately predict conversion from MCI to AD when combined with cognitive and genetic data^47^. It is likely that rational use of anti-amyloid therapies among asymptomatic individuals would be enhanced by the addition of a proximity marker based on early neural dysfunction, and our results suggest that effective connectivity could be an ideal candidate for this, especially because they demonstrate that effective connectivity can be used to make predictions, not only about who will develop dementia, but also the time until future diagnosis. These predictions were more accurate than previous prognostic models trained on structural MRI data and functional connectivity features^48^.

We acknowledge that fMRI has its limitations as a diagnostic and prognostic tool. It is expensive and the signal can be degraded in the presence of excessive head motion. This is reflected in the high exclusion rate in our own analysis. We used strict exclusion thresholds so that the models were trained on a high-quality dataset, but future work will need to assess the tolerance that these methods have for lower-quality data.

A further limitation of this study is uncertainty around how generalizable these effective connectivity-based models will be. First, our models rely on a feature selection step (Bayesian model reduction) that occurs prior to cross-validation. In theory, this might inflate model performance metrics; however, our sensitivity analysis showed that model performance was comparable when this feature selection step was omitted. Furthermore, by running Bayesian model reduction on the entire dataset, we anticipate that this simplified model is more likely to generalize to external datasets than models using the entire effective connectivity matrix. It will be crucial to validate these preliminary results on an external sample. A second point revolves around who exactly these models should be validated on. Our sample dataset is from the UKB. This cohort—comprising approximately half a million UK-based participants—is healthier than the general population^49^ and less socio-economically deprived than non-participants^50^, with a disproportionately high number of white participants. The subsample of this cohort who underwent brain imaging is younger, and has better psychological and physical health compared to the average UKB participant^51^. The generalizability of these results to a more representative sample needs to be assessed.

Another important caveat of using a UKB dataset for this study is that our labeling of cases and controls relies on clinician coding rather than standardized diagnostic criteria. Although a clinician-coded diagnosis is likely to be more clinically relevant, it may mean that symptoms of the disease were already present in the prediagnostic phase, limiting our ability to assess this as a true preclinical biomarker. Indeed, our analysis of cognitive test data revealed that the prediagnostic cases were cognitively impaired with respect to controls. Without more longitudinal cognitive data, it is not clear whether this cognitive impairment reflects cognitive decline from a pathological dementia process, or simply the baseline characteristics of this sample (we note that rates of secondary education attendance were significantly lower in the case-sample; see Extended Data Table 1). The median time to diagnosis in our prediagnostic sample was 3.7 years, and it is likely that some of these participants already had MCI. Another avenue of future research will be assessing effective connectivity-based biomarkers at even earlier stages in the pathological process, before any cognitive decline is expected to occur.

Our use of a population cohort of all-cause dementia, rather than a well phenotyped AD-specific cohort, is both a strength and a limitation of this work. Dementia is typically due to mixed pathologies, and syndromic diagnoses in life are frequently found to be incorrect at post mortem^52^. From a pragmatic population health standpoint, the ability to accurately predict all-cause dementia is therefore desirable, and makes it likely that the results of this study would be generalizable to real-world settings. We found associations between DMN effective connectivity and AD polygenic risk score, as well as between DMN effective connectivity and hippocampal and parahippocampal volumes. Although these findings suggest that these effective connectivity changes at least partially represent pathological changes specific to AD, our ability to make pathology-specific inferences is limited. Stronger evidence for a specific relationship with AD pathology could be obtained through future work incorporating biomarkers of AD proteinopathies. Indeed, in previous works, classifiers have made improved predictions on preclinical cohorts when multimodal data were used, for instance, by combining structural MRI, genetic data, cerebrospinal fluid assays and cognitive assessments^53,54^. We anticipate that, when combined with other data modalities such as amyloid beta and tau markers, effective connectivity would be likely to yield improved predictive performance.

In an exploratory analysis of modifiable risk factors, we found that social isolation had a unique and strong association with the effective connectivity changes in the dementia cohort. This finding has important implications for our understanding of why DMN dysconnectivity is so frequently observed in clinical and preclinical dementia^21^. There is a substantial overlap between the DMN and what is typically described as a social cognition network^23,55^. The mPFC, temporal poles, precuneus and temporo-parietal junction consistently activate during cognitive tasks in which participants are required to think about another person’s intentions or beliefs (that is, engage in Theory of Mind)^55–57^. There is emerging evidence that this network of brain regions is highly sensitive to one’s social environment. Social isolation has been shown to cause hypomyelination in rats^58,59^, which can be reversed through re-socialization^58,60^. In humans, childhood development of social cognitive skills is associated with white matter tract maturation in the mPFC and temporo-parietal junction^61^. In adulthood, myelin density in the mPFC is associated with one’s ability to flexibly switch between one’s own point of view and another person’s point of view^62^. Social isolation is a well-established risk factor for dementia^1,63–66^. Psychosocial interventions such as cognitive stimulation therapy can improve symptom burden^67^ and may also reverse some of the changes in DMN functional connectivity that are seen in AD^68^. These interventions are thought to weaken the link between underlying dementia pathology and cognitive decline, by promoting compensatory brain changes and expanding cognitive reserve^69^.

From a neurobiological perspective, DMN dysconnectivity is thought to be a consequence of activity-dependent tau spread, from the medial temporal lobes to densely connected cortical hubs^8,10^. Here, we found that changes in DMN effective connectivity mediated an association between social isolation and dementia incidence. This finding is consistent with a theory that social isolation triggers the DMN dysconnectivity observed in dementia. However, an important limitation of our study is that we are unable to determine which DMN effective connectivity changes are pathological and which are compensatory.

We identified multiple changes to both inhibitory and excitatory connections. Some of these connections were strengthened in dementia cases whilst others were attenuated. Examining the three largest connection differences between cases and controls, we saw a strengthening of inhibitory tone from both prefrontal and intraparietal cortices to the medial temporal lobe, and a weakening of inhibitory tone from the medial temporal lobe to the prefrontal cortex. Electrophysiological studies in people with AD show cortical hyperexcitability^70,71^, while in vivo mouse research has shown that tau silences neurons, and actually reduces excitability despite the hyperexcitability caused by amyloid beta (ref. 72). Of the three largest connection changes we observed, the only weakened connection was an inhibitory connection from the parahippocampal formation to the prefrontal cortex. This could be due the neuron-silencing effect of tau accumulation in the medial temporal lobe in the early stages of the disease. We speculate that the increased inhibitory tone from frontal and parietal DMN hubs to the medial temporal lobe may reflect a homeostatic compensation to maintain excitation–inhibition balance within the network. An important avenue for future research will be to collect longitudinal imaging data along with data on subjective and objective cognitive impairment to understand the clinical significance of different connections within the DMN dysconnectivity pattern. DMN dysconnectivity is also likely to be better understood in the context of wider cortical dynamics that involve other long-range networks. The salience network and frontoparietal control network are both altered in those at high risk of AD^73^ and these changes are associated with future cognitive decline^74^. It may be useful to include these networks in the development of future effective connectivity-based predictive models.

In summary, we found that effective connectivity in the DMN can be used as a non-invasive population-based prediagnostic biomarker for predicting future dementia incidence. This biomarker, using rs-fMRI data, is superior to using structural MRI data. The connectivity changes in the DMN are strongly associated with AD polygenic risk and social isolation, a risk factor that might accelerate the effects of pathological protein in the DMN.

## Methods

### Study design

This was a nested case-control study designed to assess whether DMN effective connectivity can be used predict two outcomes of interest. The first outcome is a future diagnosis of dementia. The second outcome is time until dementia diagnosis. Potential confounders, which we identified and tried to control for in our analyses, included age, sex, ethnicity, handedness, in-scanner head motion, geographical location of data acquisition and social deprivation (Townsend index). All statistical tests reported are two-sided.

### UKB sample selection

The UKB is a longitudinal cohort study that is regularly updated with healthcare outcomes from national UK primary and secondary healthcare databases. We identified all UKB participants who have ever had a dementia diagnosis on their health record, as of the UKB data update in May 2023, and who also had rs-fMRI data available on the UKB. Our sample size was therefore determined by data availability. Selection bias was mitigated in this study by identifying every single participant with a dementia diagnosis. This yielded an initial sample of 148 dementia cases. For each of these dementia cases, we identified ten control participants from the UKB who did not have a dementia diagnosis on their health record, and matched them with the dementia case in terms of age, sex, handedness, ethnicity and the geographical location of the MRI scanning center. After excluding participants who failed the preprocessing stage (for example, excessive head motion) and replacing failed controls with new matched controls we were left with a final usable sample of 103 cases and 1,030 matched controls. Of these 103 cases, 81 did not have a dementia diagnosis at the time of MRI data acquisition, whereas 22 already had prevalent dementia. In total, 1,486 control participants were screened through data preprocessing before the target number of 1,030 was achieved.

Participant sex identification was acquired from a central registry (the National Health Service) at the time of recruitment to the UKB, but in some cases was updated through participant self-report. Participant ethnicity was defined through self-report at the time of recruitment to the UKB. Participants were asked to report their ethnicity as ‘white’, ‘mixed’, ‘Asian or Asian British’, ‘Black or Black British’, ‘Chinese’, ‘other ethnic group’, ‘do not know’ or ‘prefer not to answer’.

### MRI data acquisition

Magnetic resonance imaging data were acquired from 2014 onwards as part of the UKB prospective cohort study, across multiple sites in the United Kingdom (Manchester, Newcastle and Reading). The scanner was a Siemens Skyra 3 T with a Siemens 32-channel RF receive head coil. Each participant underwent a 35 min scanning session, during which the following data were acquired: a T1-weighted structural image, rs-fMRI time-series, a T2-weighted FLAIR structural image, a diffusion MRI structural image, a susceptibility-weighted image and task-based fMRI time-series data. We only used the T1 image and the rs-fMRI data for our analyses.

The T1-weighted image was acquired in a 5 min 3D MPRAGE sequence with a resolution of 1 mm isotropic. The rs-fMRI data were acquired using a 6 min GE-EPI sequence with ×8 multislice acceleration. Resolution, 2.4 mm isotropic; repetition time (TR), 0.735 s; echo time (TE), 39 ms; flip angle, 52°. Data were acquired under the same protocols for cases and controls.

### MRI data preprocessing

Preprocessing was performed on raw UKB imaging data in SPM12 using batch scripts in MATLAB R2023a. First, the T1-weighted structural image was segmented into tissue subtypes, skull-stripped and then warped into Montreal Neurological Institute (MNI) space. The rs-fMRI data were spatially realigned to the single-band reference scan that was acquired in addition to the multi-band EPI sequence. Volumes were then co-registered to the skull-stripped T1 image, normalized to MNI space and spatially smoothed using a 6 mm isotropic Gaussian kernel.

In-scanner head motion was estimated for each participant by computing framewise displacement for each participant using the three translational and three rotational motion parameters (assuming rotation around the surface of a sphere with radius 50 mm). Participants were excluded from further analysis if their maximum framewise displacement exceeded 2.4 mm. This threshold was chosen because it was the voxel resolution of the dataset.

### Time-series extraction

A DMN was constructed by pre-defining ten ROIs on the basis of pre-existing literature^7,75^. This number of ROIs was chosen to compromise between anatomical detail and feasible computation time when fitting dynamic causal models. The ten-node network comprised a core DMN of the anterior medial prefrontal cortex (amPFC), the precuneus, and the left and right intraparietal cortex (IPC). These four ROIs were centered around the following co-ordinates, respectively, on the basis of a previous study on DMN effective connectivity by Almgren and colleagues^75^: (*x* = 2, *y* = 56, *z* = –4), (*x* = 2, *y* = –58, z = 30), (*x* = –44, *y* = –60, *z* = 24), (*x* = 54, *y* = –62, *z* = 28). We included the following additional ROIs in our DMN network, using co-ordinates from a past study on DMN connectivity in amnestic cognitive impairment by Dunn and colleagues^7^: ventromedial prefrontal cortex (vmPFC), dorsomedial prefrontal cortex (dmPFC), left and right lateral temporal cortex (LTC) and left and right parahippocampal formation (PHF), centered on the following co-ordinates, respectively: (*x* = 0, *y* = 26, *z* = 18), (*x* = 0, *y* = 52, *z* = 26), (*x* = –60, *y* = –24, *z* = 18), (*x* = 60, *y* = –24, *z* = 18), (*x* = –28, *y* = –40, *z* = –12), (*x* = 28, *y* = –40, *z* = –12).

The signal from each ROI was estimated by fitting a general linear model containing a discrete cosine basis set with frequency range 0.0078–0.1 Hz, as well as the following nuisance regressors: six head motion regressors, a regressor for cerebrospinal fluid signal (a principal eigenvariate sphere radius of 5 mm centered in the third ventricle at (*x* = 0, *y* = –40, *z* = –5)), a regressor for white matter signal (a principal eigenvariate sphere radius of 6 mm centered in the brainstem at (*x* = 0, *y* = –24, z = –33)). Global signal regression was not performed as there is evidence it does not substantially impact results in small network analyses^75^. An F-contrast was specified across all components of the discrete cosine basis set, yielding a BOLD time-series of low-amplitude fluctuations in each voxel within a 10 mm radius sphere centered on each of the ten ROI co-ordinates listed above.

For each ROI, a new 8 mm sphere was then centered on the peak intensity voxel. A summary signal for the ROI was computed as the principal eigenvariate of all supra-threshold voxels (uncorrected α = 0.05) that lay in the conjunction space of the first 10 mm sphere and the second 8 mm sphere. These were voxels with evidence for low frequency BOLD fluctuations. Note that the principal eigenvariate across voxels is used, rather than the mean, so that negative and positive signals do not negate each other and that the extreme values don’t bias the mean estimate. If any of the ten ROIs yielded no supra-threshold voxels then the participant was excluded from further analysis.

### Estimating effective connectivity

Effective connectivity was estimated using spectral DCM using the DCM12 toolbox in SPM12. Spectral DCM fits a biophysical state-space model to the observed cross-spectra of BOLD signals, to estimate underlying neuronal states^30^ and the rate of change in neural activity in each region (in hertz) as a function of activity in other regions (that is effective connectivity). For each participant we fitted a fully connected DCM with a connectivity parameter for every possible pair of the ten ROIs, including auto-inhibitory self-connections. This model thus comprised 100 connectivity parameters. The DCM software^76,77^ uses the variational Laplace algorithm to invert the model and estimate these connectivity parameters by minimizing negative free energy. We used the software’s default priors. Each participant’s DCM fit was screened for convergence by ensuring it met the following criteria: explained variance of BOLD signal greater than 10%, at least one connection (excluding self-connections) with an absolute connection strength of greater than 1/8 Hz, and at least one effectively estimated parameter (based on the Kullback–Leibler divergence of posterior from prior). All participants who had a DCM fitted met these criteria for model convergence.

We fit a parametric empirical Bayes (PEB) model^78^ to the full set of participant-specific DCMs to estimate an average connectivity matrix across participants and estimate the difference in connectivity between cases and controls. The PEB technique enables us to estimate group-level connectivity strengths by fitting a hierarchical model to the estimated connectivity parameters of each individual and the precisions of those parameters. We specified a between-participants design matrix that contained five columns: a column of ones, to model the average connectivity strengths across all participants; a column of ones and zeros, to model the differences in connectivity between cases and controls; and three columns to model covariates of no-interest (age, sex and mean framewise displacement to model any effects attributable to head motion). The last three columns were mean-centered. Instead of estimating a full covariance matrix across connectivity parameters, a single precision component was shared across connectivity parameters, to permit model estimation within a reasonable amount of time. The resulting PEB model comprised 500 connectivity parameters, that is, a 10 × 10 connectivity matrix for each of the five columns of the between-participants design matrix.

Finally, we used exploratory Bayesian model reduction and Bayesian model comparison to find the best (and simplest) model to explain the data. In this procedure an automatic greedy search over reduced models iteratively discards parameters that don’t contribute to model evidence. A Bayesian model average of parameters is then calculated over the 256 models from the final iteration of the greedy search (default settings of DCM software).

The details of the biophysical model used in DCM, model inversion at a participant and group level and Bayesian model reduction have already been extensively documented^76,77,79^ and will not be reproduced here.

### Case-control classifier

Of the group-level parameters that model differences in effective connectivity between cases and controls, we selected all parameters with an at least 99% posterior probability of being non-zero. This way we identified a set of statistically plausible connections to use as data features for our classifier.

We trained an elastic-net regularized logistic regression model on these features to classify cases from controls using the glmnet toolbox for MATLAB. To accommodate for the 10:1 imbalance in class size, observation weights were applied so that cases were weighted ten times more than controls. A nested stratified *k*-fold cross-validation (CV) scheme was applied for tuning two hyperparameters: elastic mixing parameter *α* and regularization penalty *λ*.

The dataset was partitioned into *K* = 10 subsets. The first subset contained the 22 participants with prevalent dementia at the time of data acquisition as well as their 220 matched controls; the remaining nine contained a random sampling of the rest of the dataset, with the requirement that each subset contained ten controls per case.

For each outer fold of CV, one subset was held out as a test set while the remaining nine subsets constituted a train-set. Note that the first subset was never used as a test set and only nine folds of outer cross-validation were actually performed. Therefore, data from the 22 participants who had a prevalent diagnosis of dementia at the time of data acquisition were only used to train the model, whereas the performance of the model exclusively refers to its ability to predict a future dementia diagnosis in those who did not yet have a diagnosis at the time of data acquisition.

For each of these nine outer folds of CV, the train-set was randomly partitioned into *K* = 5 inner subsets, again with the requirement that each inner subset contained ten controls per case. Thus, five folds of inner CV were performed. Each of these five inner folds was repeated for a different value of *α*, ranging from zero to one in increments of 0.1. Glmnet automatically uses a range of 100 *λ* values every time a model is estimated. A different ROC curve was generated for each possible combination of hyperparameters, and for each inner fold of CV. The AUC was averaged across the five inner folds. The combination of hyperparameters that generated the maximum average AUC were then used for a model trained on the entire train-set and applied to the originally held out test set. At the end of the procedure, nine AUC curves were generated, one for each outer fold of CV. The mean AUC from these nine ROC curves was used as the final AUC.

As *K*-fold CV is sensitive to the way that the data is partitioned, the entire procedure described above was performed nine times, with a different random partitioning of data each time. The median AUC from these nine iterations is reported as the main result with the minimum and maximum AUC reported in brackets. The ROC curves from all nine iterations are plotted in Fig. 4.

The above analysis was also performed using nested leave-one-out CV instead of *K*-fold CV, to generate a robust and unique participant-specific probability of dementia. We call this participant-specific value ‘effective connectivity (EC) index’ and it was used for subsequent analyses on individual differences (see the ‘Volumetric data analysis’ and ‘Modifiable risk factors analysis’ sections below).

### Prognosticator

We trained a prognosticator model to test whether effective connectivity features could also be used to predict when these individuals got their dementia diagnosis. A group-level effective connectivity matrix was computed, using the PEB framework with Bayesian model reduction, as described above, but this time only the dementia cases were included in the analysis. The second column in the between-participants design matrix was not a column of ones and zeros to represent cases and controls, but rather a continuous variable that was computed as date of MRI acquisition subtracted from the date of dementia diagnosis (that is, how long, in years, until a dementia diagnosis). The value was negative if the participant already had a dementia diagnosis at the time of data acquisition. One participant, with prevalent dementia at the time of data collection, was excluded from this analysis as there was no reliable date of their past dementia diagnosis. Of the group-level parameters that model differences in effective connectivity as a function of the time until diagnosis, we selected all parameters with a posterior probability of at least 99% of being non-zero.

We then trained an elastic-net regularized linear regression model using the same *K*-fold cross-validation scheme as described above for the classifier. However, in this analysis, we wanted to assess the ability of the prognosticator to predict both positive and negative time until dementia diagnosis. The model was therefore tested on the cases with prevalent dementia at the time of data acquisition, and therefore all *K* = 10 subsets were used as test sets and ten folds of outer CV were performed. Hyperparameters *α* and *λ* were tuned by minimizing the squared error between predictions and true values. Performance was evaluated as the Spearman correlation coefficient between final model predictions are true values. As above, the entire procedure was iterated nine times, the final reported result was the median Spearman coefficient across the nine iterations.

### Volumetric data analysis

We repeated the above analyses to see how useful effective connectivity parameters were at making predictions about dementia compared to other MRI-based features, but this time using volumetric data features from structural MRI instead. We used pre-existing volume data from UKB’s imaging-derived phenotype database^80^. Specifically, we used the following 18 hippocampal subsegmental volumes (segmented using FreeSurfer): body of CA1, head of CA1, body of CA3, head of CA3, body of CA4, head of CA4, molecular layer of hippocampal body, molecular layer of hippocampal head, parasubiculum, presubiculum body, presubiculum head, subiculum body, subiculum head, whole hippocampal tail, whole hippocampal body, whole hippocampal head, whole hippocampus and hippocampal fissure. We also used two additional gray matter volumes from UKB’s imaging-derived phenotype database, segmented using FMRIB’s automated segmentation tool (FAST): anterior division of parahippocampal gyrus and posterior division of parahippocampal gyrus. Volumes were used from both the left and right hemispheres, and thus a total of 40 features were used.

Each feature was normalized by total intracranial volume. We then trained regularized logistic regression and linear regression models on this volumetric data using exactly the same cross-validation procedures that we used for the effective connectivity data features, as described above.

We also tested for an association between effective connectivity index and volumetric data. We took the mean across all 40 subsegmental volumes and fit a weighted linear regression model using fitglm in MATLAB to see whether average volume was associated with effective connectivity index. Individuals with dementia were upweighted and control participants were downweighed such that cases and controls made equal contributions to the regression model. We then ran 40 separate post-hoc regressions where the predictor variable was each individual subsegmental volume. Only the regression models that yielded the three highest *R*^2^ values are reported.

### Functional connectivity analysis

We estimated functional connectivity matrices for each participant to compare predictions based on effective connectivity to an alternative rs-fMRI metric. For this analysis we used the same BOLD time-series that were used for the DCM analysis. For each possible pair of ROIs, a Fisher *z*-transformed Pearson correlation coefficient was computed between the BOLD time-series from these two ROIs. This generated a 10 × 10 functional connectivity matrix for each participant. As this matrix is symmetrical, duplicate elements were removed and the diagonal elements (self-connections) were also removed. This resulted in 45 functional connectivity values. We then trained regularized logistic regression and linear regression models on these functional connectivity values using exactly the same cross-validation procedures that we used for the effective connectivity data features, as described above.

### Cognitive data analysis

To assess the cognitive profile of the cases and controls in this study, we utilized UKB data from touchscreen cognitive function tests. Multiple cognitive tests were performed but only four tests were deemed to have sufficient data for analysis. For the other cognitive tests, at least 30% of our analyzed participants had missing data. The four cognitive tests that we used for analysis were assessments of visual declarative memory, processing speed, verbal and numerical reasoning and prospective memory. Missing data were imputed with the median across all participants. Details of the cognitive tests and performance of cases and controls in each of the four tests can be seen in Supplementary Table 3.

We found significant differences between cases and controls in reaction time, fluid intelligence and prospective memory, with controls performing better in all three tasks. To assess how well these cognitive data could predict future dementia diagnosis and time until dementia diagnosis, we trained regularized logistic regression and linear regression models on these cognitive outcome measures using exactly the same cross-validation procedures that we used for the effective connectivity data features, as described above.

We also constructed a composite score of cognitive ability by running a principal components analysis on the four test scores. We took individual scores for the first principal component, which loaded negatively on number of errors in the pairs matching test and reaction time, and loaded positively on scores in the fluid intelligence and prospective memory task (that is, a higher score on this principal component indicated better performance across the four tasks).

### Modifiable risk factors analysis

We investigated which modifiable risk factors were associated with dementia-related changes in DMN effective connectivity using multiple multivariable linear regression models. We constructed a variable for each of the 12 modifiable risk factors identified in the 2020 Lancet commission on dementia^1^. History of hypertension, diabetes, smoking, depression, physical inactivity, traumatic brain injury and hearing loss, absence of secondary education, and residence in a highly polluted neighborhood (top decile) were coded as binary variables. Body mass index, weekly alcohol consumption and social isolation were coded as continuous numerical variables. The social isolation variable was constructed with data from three questions, which participants answered as part of the touchscreen session at baseline data collection. These three questions assessed: (1) weekly attendance at social leisure activities (binary); (2) an estimated number of visits from friends or family within a year (continuous numerical); and (3) an estimated number of times the participant felt able to confide in someone close to them within a year (continuous numerical). We ran a principal components analysis on these three variables and took individual scores for the first principal component, which loaded negatively on all three variables (that is, a higher score on this principal component indicated greater social isolation). Traumatic brain injury was excluded from the subsequent regression analyses as there were only nine positive cases across the entire sample. This left 11 modifiable risk factors for analysis. For all variables, missing data were imputed with the median across all participants (see Supplementary Table 2 for numbers of missing data points). Data acquisition and processing were identical for cases and controls. Supplementary Table 4 shows details of the raw UKB variables used to derive the variables in this analysis.

For each of the 11 modifiable risk factors, as well as AD PRS, a weighted linear regression model was estimated using fitglm in MATLAB, where the risk factor of interest was the predictor variable, and effective connectivity index was the response variable. The effective connectivity index is simply the probability of dementia outputted from the case-control classifier trained with leave-one-out cross-validation. A higher value here indicates that the participant’s over-all effective connectivity pattern conforms more to a dementia-like phenotype than a control-like phenotype. Age, sex and Townsend social deprivation score were included as covariates of no-interest in each of the 12 linear regression models. Participants with dementia were upweighted and control participants were downweighed in the linear regression models, such that cases and controls made equal contributions to the regression models. A *P*-value was estimated for each of the 11 modifiable risk factors and for PRS, which was corrected for multiple comparisons using the Holm–Bonferroni method, to maintain a family wise error rate of 0.05.

A mediation analysis was performed, with social isolation as a predictor, effective connectivity index as a mediator and dementia incidence as a response variable (dummy-coded binary variable). Each regression model estimated in the mediation analysis included age, sex and Townsend social deprivation score as covariates of no-interest, and used weighted observations such that cases and controls contributed equally to the model. A *P*-value was estimated for the significance of the indirect path coefficient by generating a permutation-based null distribution. For each permutation, the dementia incidence variable was randomly shuffled and an indirect path coefficient was estimated. This was repeated 1,000 times to generate a null distribution of indirect path coefficients with which to evaluate the true indirect path coefficient magnitude.

### Alzheimer’s disease polygenic risk score

The AD PRS was downloaded from the UKB standard PRS set^81^. The database comprises PRSs for 28 diseases and 25 traits for every UKB participant. Polygenic risk scores were derived from meta-analysis of multiple external genome-wide association study sources. Detailed methods for how PRSs were generated have already been extensively documented^81^ and will not be reproduced here.

### Ethics and inclusion statement

This research included local researchers throughout the research process. Roles and responsibilities were agreed amongst collaborators ahead of the research. This research involved no health, safety, security or other risk to participants or researchers.

### Data access and ethics

This research was conducted using the UKB Resource under application no. 78867 (PI: C. Marshall). Informed written consent was obtained from all participants on enrollment in UKB and they were informed that they are free to withdraw their consent at any time, at which time their data would be censored and excluded from future analysis. Participants were offered compensation for reasonable travel expenses. The UKB has approval from the North West Multicentre Research Ethics Committee as a Research Tissue Bank (REC reference: 21/NW/0157).

## Reporting summary

Further information on research design is available in the Nature Portfolio Reporting Summary linked to this article.

## Extended Data

**Extended Data Table 1:** Sample characteristics

Characteristic	Cases	Controls	P
Age, y(SD)	70.63 (6.7)	70.19 (6.58)	0.52 (t-test)
Women, N (%)	44 (43%)	428 (42%)	0.82 (chi squared)
Men, N (%)	59 (57%)	602 (58%)	0.82 (chi squared)
Ethnicity, N white (%)	102 (99%)	1023 (99%)	0.74 (chi squared)
Ethnicity, N black (%)	1 (1%)	7 (1 %)	0.74 (chi squared)
Right handed, %	93	96	0.24 (chi squared)
Median Social isolation score	0.3	-0.003	**0.0026** (Mann-Whitney U test)
Hearing loss, %	67	62.2	0.34 (chi squared)
Pollution, %	9.7	8.3	0.64 (chi squared)
Smoking, %	53.4	58	0.37 (chi squared)
Depression, %	21.4	12	**0.007** (chi squared)
Hypertension, %	50.5	41.6	0.08 (chi squared)
Median alcohol intake	52.1	52.1	0.95 (Mann-Whitney U test)
Less education, %	15.5	8.2	0.01 (chi squared)
Diabetes, %	10.7	5.9	0.06 (chi squared)
Physical inactivity, %	63.1	62.6	0.92 (chi squared)
Median Body mass index (BMI)	25.8	25.9	0.48 (Mann-Whitney U test)
Social deprivation score	-2.95	-2.77	0.74 (Mann-Whitney U test)

Breakdown of sample demographics for cases and controls. *P*-values are shown for statistical tests for differences in demographic numbers between cases and controls. All statistical tests are two-sided.

## Supplementary Material

**Supplementary information** The online version contains supplementary material available at https://doi.org/10.1038/s44220-024-00259-5.

Supplementary Material

Reporting Summary

## Data Availability

Processed group-level DCM results are available at https://github.com/Wolfson-PNU-QMUL/UKB_DCM_dementia. Supplementary Table 4 contains UKB field names for UKB data variables analyzed in this study.

## Code availability

MATLAB analysis code is available at https://github.com/Wolfson-PNU-QMUL/UKB_DCM_dementia.
