# Supplementary material for: Early detection of dementia with default-mode network effective connectivity
Source: Nat Ment Health. Author manuscript; Available in PMC 2026 Feb 18. (PMC7618740; doi:10.1038/s44220-024-00259-5)
Supplement: Supplementary Material [file EMS212461-supplement-Supplementary_Material.zip › 44220_2024_259_MOESM1_ESM.pdf]

nature mental health

Supplementary information https://doi.org/10.1038/s44220-024-00259-5

Early detection of dementia with default-mode network effective connectivity

In the format provided by the authors and unedited

RISK FACTOR	BETA	SE	P (UNCORRECTED)
POLYGENIC RISK SCORE (PRS)	0.053	0.0076	3.7×10^{-12}
SOCIAL ISOLATION	0.0247	0.0082	0.003
HEARING LOSS	0.0179	0.0085	0.035
POLLUTION	0.0119	0.0084	0.16
SMOKING	0.0110	0.0084	0.19
DEPRESSION	0.0089	0.0076	0.24
HYPERTENSION	0.0095	0.0086	0.27
ALCOHOL	0.0084	0.0082	0.30
LESS EDUCATION	– 0.0078	0.0076	0.30
DIABETES	– 0.0059	0.0075	0.43
PHYSICAL INACTIVITY	– 0.0032	0.0084	0.70
BODY MASS INDEX (BMI)	– 0.0019	0.0084	0.82

Table S1 – Regression results for analysis of relationship between effective connectivity index and various risk factors. All statistical tests are two-sided.

VARIABLE	N MISSING (%)
POLYGENIC RISK SCORE (PRS)	1 (0.09)
FREQUENCY OF VISITS FROM FRIENDS AND FAMILY	14 (1.24)
ENGAGEMENT IN SOCIAL ACTIVITIES	12 (1.06)
FREQUENCY OF CONFIDING	52 (4.6)
HEARING LOSS	0 (0)
POLLUTION	59 (5.21)
SMOKING	19 (1.68)
DEPRESSION	0 (0)
HYPERTENSION	0 (0)
ALCOHOL	92 (8.12)
LESS EDUCATION	0 (0)
DIABETES	0 (0)
PHYSICAL INACTIVITY	167 (14.74)
BODY MASS INDEX (BMI)	29 (2.56)
PAIRS MATCHING TEST	100 (8.83)
REACTION TIME TEST	121 (10.68)
VERBAL AND NUMERICAL REASONING TEST	140 (12.36)
PROSPECTIVE MEMORY TEST	101 (8.91)

Table S2 – Number of participants with missing data for each analysed variable, also expressed in brackets as a percentage of the total analysed sample (N = 1133)

TEST	COGNITIVE DOMAIN	OUTCOME MEASURE	PREVALENT CASES (N = 22)	INCIDENT CASES (N = 81)	CONTROLS (N = 1030)	P (INCIDENT CASES VS. CONTROLS)
PAIRS MATCHING	Visual declarative memory	Mean number of errors	4.4	4.1	3.9	0.89 (Mann-Whitney U test)
REACTION TIME	Processing speed	Median reaction time (ms)	616	611	597	**0.01** (Mann-Whitney U test)
FLUID INTELLIGENCE	Verbal and numerical reasoning	Mean score out of 13	5.5	5.9	6.7	**0.002** (Mann-Whitney U test)
PROSPECTIVE MEMORY	Memory	% accurate participants	59%	68%	81%	**0.003** (chi squared)

Table S3 – Performance of cases and controls across four cognitive tests

The final column indicates the P value of a statistical test of the difference in score between incident cases and controls. All statistical tests are two-sided.

Pairs Matching: The participant is asked to memorise the position of as many matching pairs of cards as possible. The cards are then turned face down on the screen and the participant is asked to touch as many pairs as possible in the fewest tries.

Reaction time: The participant is shown two cards at a time; if both cards are the same, they press a button-box that is on the table in front of them as quickly as possible.

Fluid intelligence: The participant is asked a series of verbal and numerical reasoning questions. The specific questions can be seen here: https://biobank.ndph.ox.ac.uk/showcase/ukb/docs/Fluidintelligence.pdf.

Prospective memory: Early in the touchscreen cognitive section, the participant is shown the message "At the end of the games we will show you four coloured shapes and ask you to touch the Blue Square. However, to test your memory, we want you to actually touch the Orange Circle instead." This assessment yields a single binary correct or incorrect, depending on how the participant responds when shown the four coloured shapes at the end of the assessment.

Table S4 – UK Biobank field names for variables analysed

Exposure	Raw UKB variables (variable names are derived from UKBTools)
**Dementia**	**date_of_all_cause_dementia_report_f42018_0_0**<br>**date_of_frontotemporal_dementia_report_f42024_0_0**<br>**date_f02_first_reported_dementia_in_other_diseases_classified_elsewhere_f130840_0_0**<br>**date_f03_first_reported_unspecified_dementia_f130842_0_0**<br>**date_of_alzheimers_disease_report_f42020_0_0**<br>**date_f00_first_reported_dementia_in_alzheimers_disease_f130836_0_0**<br>**date_g30_first_reported_alzheimers_disease_f131036_0_0**<br>**date_of_vascular_dementia_report_f42022_0_0**<br>**date_f01_first_reported_vascular_dementia_f130838_0_0**<br>**ICD10: diagnoses_icd10_f41270_0_0** through **diagnoses_icd10_f41270_0_258 (259 arrays)** using these ICD10 codes (**date_of_first_inpatient_diagnosis_icd10_f41280_0_0** through **date_of_first_inpatient_diagnosis_icd10_f41280_0_258** was used to define the date):<br>F00 F00 Dementia in Alzheimer's disease<br>F000 F00.0 Dementia in Alzheimer's disease with early onset<br>F001 F00.1 Dementia in Alzheimer's disease with late onset<br>F002 F00.2 Dementia in Alzheimer's disease, atypical or mixed type<br>F009 F00.9 Dementia in Alzheimer's disease, unspecified<br>G300 G30.0 Alzheimer's disease with early onset<br>G301 G30.1 Alzheimer's disease with late onset<br>G308 G30.8 Other Alzheimer's disease<br>G309 G30.9 Alzheimer's disease, unspecified<br>F01 F01 Vascular dementia<br>F010 F01.0 Vascular dementia of acute onset<br>F011 F01.1 Multi-infarct dementia<br>F012 F01.2 Subcortical vascular dementia<br>F013 F01.3 Mixed cortical and subcortical vascular dementia<br>F018 F01.8 Other vascular dementia<br>F019 F01.9 Vascular dementia, unspecified<br>A810 A81.0 Creutzfeldt-Jakob disease<br>F02 Dementia in other diseases classified elsewhere<br>F020 F02.0 Dementia in Pick's disease<br>F021 F02.1 Dementia in Creutzfeldt-Jakob disease<br>F022 F02.2 Dementia in Huntington's disease<br>F023 F02.3 Dementia in Parkinson's disease<br>F024 F02.4 Dementia in human immunodeficiency virus [HIV] disease<br>F028 F02.8 Dementia in other specified diseases classified elsewhere<br>F03 F03 Unspecified dementia<br>F051 F05.1 Delirium superimposed on dementia<br>G310 G31.0 Circumscribed brain atrophy<br>G311 G31.1 Senile degeneration of brain, not elsewhere classified<br>G318 G31.8 Other specified degenerative diseases of nervous system<br>I673 I67.3 Progressive vascular leukoencephalopathy (Binswager)<br><br>(binary definition derived across all of these fields, using the associated date fields to obtain the earliest diagnosis)
**Age**	**age_when_attended_assessment_centre_f21003_2_0**
**Sex**	**sex_f31_0_0**
**Handedness**	**handedness_chiralitylaterality_f1707_2_0**
**Ethnicity**	**ethnic_background_f21000_0_0**
**Social Deprivation (Townsend Index)**	**townsend_deprivation_index_at_recruitment_f22189_0_0**
**Frequency of Visits from Friends and Family**	**frequency_of_friendfamily_visits_f1031_2_0**
**Engagement in Social Activities**	**leisuresocial_activities_f6160_2_0** through **leisuresocial_activities_f6160_2_4**
**Frequency of Confiding**	**able_to_confide_f2110_2_0**
**Hearing Loss**	**hearing_difficultyproblems_f2247_2_0**<br>**hearing_difficultyproblems_with_background_noise_f2257_2_0**<br>**speechreceptionthreshold_srt_estimate_left_f20019_2_0**<br>**speechreceptionthreshold_srt_estimate_right_f20021_2_0**<br>**date_h90_first_reported_conductive_and_sensorineural_hearing_loss_f131258_0_0**

	**date_h91_first_reported_other_hearing_loss_f131260_0_0**<br>**date_h93_first_reported_other_disorders_of_ear_not_elsewhere_classified_f131264_0_0**<br>**date_h94_first_reported_other_disorders_of_ear_in_diseases_classified_elsewhere_f131266_0_0**<br>**date_h95_first_reported_postprocedural_disorders_of_ear_and_mastoid_process_not_elsewhere_classified_f131268_0_0**<br>**ICD10: diagnoses_icd10_f41270_0_0** through **diagnoses_icd10_f41270_0_258 (259 arrays)** using these ICD10 codes (**date_of_first_inpatient_diagnosis_icd10_f41280_0_0** through **date_of_first_inpatient_diagnosis_icd10_f41280_0_258** was used to define the date): :<br>H90 H90 Conductive and sensorineural hearing loss<br>H900 H90.0 Conductive hearing loss, bilateral<br>H901 H90.1 Conductive hearing loss, unilateral with unrestricted hearing on the contralateral side<br>H902 H90.2 Conductive hearing loss, unspecified<br>H903 H90.3 Sensorineural hearing loss, bilateral<br>H904 H90.4 Sensorineural hearing loss, unilateral with unrestricted hearing on the contralateral side<br>H905 H90.5 Sensorineural hearing loss, unspecified<br>H906 H90.6 Mixed conductive and sensorineural hearing loss, bilateral<br>H907 H90.7 Mixed conductive and sensorineural hearing loss, unilateral with unrestricted hearing on the contralateral side<br>H908 H90.8 Mixed conductive and sensorineural hearing loss, unspecified<br>H91 H91 Other hearing loss<br>H910 H91.0 Ototoxic hearing loss<br>H911 H91.1 Presbycusis<br>H912 H91.2 Sudden idiopathic hearing loss<br>H913 H91.3 Deaf mutism, not elsewhere classified<br>H918 H91.8 Other specified hearing loss<br>H919 H91.9 Hearing loss, unspecified<br>H930 H93.0 Degenerative and vascular disorders of ear<br>H931 H93.1 Tinnitus<br>H932 H93.2 Other abnormal auditory perceptions<br>H933 H93.3 Disorders of acoustic nerve<br>H940 H94.0 Acoustic neuritis in infectious and parasitic diseases classified elsewhere<br><br>(binary definition including self-reporting hearing difficulty, ‘poor’ hearing on SRT (>= -3.5 dB), or having a clinical diagnosis of hearing deficit before the date of MRI scan)
**Pollution**	**particulate_matter_air_pollution_pm25_2010_f24006_0_0**<br><br>(top decile vs rest)
**Smoking**	**smoking_status_f20116_2_0**
**Depression**	**bipolar_and_major_depression_status_f20126_0_0**<br>**probable_recurrent_major_depression_moderate_f20124_0_0**<br>**probable_recurrent_major_depression_severe_f20125_0_0**<br>**single_episode_of_probable_major_depression_f20123_0_0**<br>**frequency_of_depressed_mood_in_last_2_weeks_f2050_2_0** (every day or >half of days taken as depression)<br>**date_f32_first_reported_depressive_episode_f130894_0_0**<br>**date_f33_first_reported_recurrent_depressive_disorder_f130896_0_0**<br>**ICD10: diagnoses_icd10_f41270_0_0** through **diagnoses_icd10_f41270_0_258 (259 arrays)** using these ICD10 codes (**date_of_first_inpatient_diagnosis_icd10_f41280_0_0** through **date_of_first_inpatient_diagnosis_icd10_f41280_0_258** was used to define the date): :<br>F32 F32 Depressive episode<br>F320 F32.0 Mild depressive episode<br>F321 F32.1 Moderate depressive episode<br>F322 F32.2 Severe depressive episode without psychotic symptoms<br>F323 F32.3 Severe depressive episode with psychotic symptoms<br>F328 F32.8 Other depressive episodes<br>F329 F32.9 Depressive episode, unspecified<br>F33 F33 Recurrent depressive disorder<br>F330 F33.0 Recurrent depressive disorder, current episode mild<br>F331 F33.1 Recurrent depressive disorder, current episode moderate<br>F332 F33.2 Recurrent depressive disorder, current episode severe without psychotic symptoms<br>F333 F33.3 Recurrent depressive disorder, current episode severe with psychotic symptoms<br>F334 F33.4 Recurrent depressive disorder, currently in remission<br>F338 F33.8 Other recurrent depressive disorders<br>F339 F33.9 Recurrent depressive disorder, unspecified<br>**Noncancer_illness_code_selfreported_f20002_0_0** through **noncancer_illness_code_selfreported_f20002_0_33 (34 arrays)** using these codes:<br>1286 depression<br>1290 deliberate self-harm/suicide attempt
**Hypertension**	**vascularheart_problems_diagnosed_by_doctor_f6150_2_0** through **vascularheart_problems_diagnosed_by_doctor_f6150_2_3**<br>**age_high_blood_pressure_diagnosed_f2966_2_0**

	**medication_for_cholesterol_blood_pressure_diabetes_or_take_exogenous_hormones_f6153_2_0** through<br>**medication_for_cholesterol_blood_pressure_diabetes_or_take_exogenous_hormones_f6153_2_3**<br>**medication_for_cholesterol_blood_pressure_or_diabetes_f6177_2_0** through<br>**medication_for_cholesterol_blood_pressure_or_diabetes_f6177_2_2**<br>**date_i10_first_reported_essential_primary_hypertension_f131286_0_0**<br>**date_i11_first_reported_hypertensive_heart_disease_f131288_0_0**<br>**date_i12_first_reported_hypertensive_renal_disease_f131290_0_0**<br>**date_i13_first_reported_hypertensive_heart_and_renal_disease_f131292_0_0**<br>**date_i15_first_reported_secondary_hypertension_f131294_0_0**<br>**ICD10: diagnoses_icd10_f41270_0_0** through **diagnoses_icd10_f41270_0_258 (259 arrays)** using these ICD10 codes (**date_of_first_inpatient_diagnosis_icd10_f41280_0_0** through **date_of_first_inpatient_diagnosis_icd10_f41280_0_258** was used to define the date): :<br>I10 I10 Essential (primary) hypertension<br>I11 I11 Hypertensive heart disease<br>I110 I11.0 Hypertensive heart disease with (congestive) heart failure<br>I119 I11.9 Hypertensive heart disease without (congestive) heart failure<br>I12 I12 Hypertensive renal disease<br>I120 I12.0 Hypertensive renal disease with renal failure<br>I129 I12.9 Hypertensive renal disease without renal failure<br>I13 I13 Hypertensive heart and renal disease<br>I130 I13.0 Hypertensive heart and renal disease with (congestive) heart failure<br>I131 I13.1 Hypertensive heart and renal disease with renal failure<br>I132 I13.2 Hypertensive heart and renal disease with both (congestive) heart failure and renal failure<br>I139 I13.9 Hypertensive heart and renal disease, unspecified<br>I15 I15 Secondary hypertension<br>I150 I15.0 Renovascular hypertension<br>I151 I15.1 Hypertension secondary to other renal disorders<br>I152 I15.2 Hypertension secondary to endocrine disorders<br>I158 I15.8 Other secondary hypertension<br>I159 I15.9 Seconday hypertension, unspecified<br>**Noncancer_illness_code_selfreported_f20002_0_0** through<br>**noncancer_illness_code_selfreported_f20002_0_33 (34 arrays)** using these codes (**interpolated_age_of_participant_when_noncancer_illness_first_diagnosed_f20009_0_0** through **interpolated_age_of_participant_when_noncancer_illness_first_diagnosed_f20009_0_33** was used to assign the age at diagnosis)::<br>1065 hypertension<br>1072 essential hypertension<br><br>(binary definition across any reports of having high blood pressure or taking medication for it in the phenotype data or having and the medical codes before the date of the MRI scan)
**Alcohol Intake**	**average_weekly_beer_plus_cider_intake_f1588_2_0**<br>**average_weekly_fortified_wine_intake_f1608_2_0**<br>**average_weekly_red_wine_intake_f1568_2_0**<br>**average_weekly_champagne_plus_white_wine_intake_f1578_2_0**<br>**average_weekly_spirits_intake_f1598_2_0**<br>**average_weekly_intake_of_other_alcoholic_drinks_f5364_2_0**<br>**average_monthly_beer_plus_cider_intake_f4429_2_0**<br>**average_monthly_fortified_wine_intake_f4451_2_0**<br>**average_monthly_intake_of_other_alcoholic_drinks_f4462_2_0**<br>**average_monthly_red_wine_intake_f4407_2_0**<br>**average_monthly_spirits_intake_f4440_2_0**<br>**average_monthly_champagne_plus_white_wine_intake_f4418_2_0**<br><br>(each of these scores was converted to alcoholic units and the sum was taken)
**Education**	**age_completed_full_time_education_f845_0_0**<br>**qualifications_f6138_0_0**<br>(no secondary education vs having secondary or greater education)
**Diabetes**	**diabetes_diagnosed_by_doctor_f2443_2_0**<br>**age_diabetes_diagnosed_f2976_2_0**<br>**medication_for_cholesterol_blood_pressure_diabetes_or_take_exogenous_hormones_f6153_2_0** through<br>**medication_for_cholesterol_blood_pressure_diabetes_or_take_exogenous_hormones_f6153_2_3**<br>**medication_for_cholesterol_blood_pressure_or_diabetes_f6177_2_0** through<br>**medication_for_cholesterol_blood_pressure_or_diabetes_f6177_2_2**<br>**date_e10_first_reported_insulindependent_diabetes_mellitus_f130706_0_0**<br>**date_e11_first_reported_noninsulindependent_diabetes_mellitus_f130708_0_0**<br>**date_e12_first_reported_malnutritionrelated_diabetes_mellitus_f130710_0_0**<br>**date_e13_first_reported_other_specified_diabetes_mellitus_f130712_0_0**<br>**date_e14_first_reported_unspecified_diabetes_mellitus_f130714_0_0**<br>**ICD10: diagnoses_icd10_f41270_0_0** through **diagnoses_icd10_f41270_0_258 (259 arrays)** using these ICD10 codes (**date_of_first_inpatient_diagnosis_icd10_f41280_0_0** through **date_of_first_inpatient_diagnosis_icd10_f41280_0_258** was used to define the date): :

	E10 E10 Insulin-dependent diabetes mellitus<br>E100 E10.0 With coma<br>E101 E10.1 With ketoacidosis<br>E102 E10.2 With renal complications<br>E103 E10.3 With ophthalmic complications<br>E104 E10.4 With neurological complications<br>E105 E10.5 With peripheral circulatory complications<br>E106 E10.6 With other specified complications<br>E107 E10.7 With multiple complications<br>E108 E10.8 With unspecified complications<br>E109 E10.9 Without complications<br>E11 E11 Non-insulin-dependent diabetes mellitus<br>E110 E11.0 With coma<br>E111 E11.1 With ketoacidosis<br>E112 E11.2 With renal complications<br>E113 E11.3 With ophthalmic complications<br>E114 E11.4 With neurological complications<br>E115 E11.5 With peripheral circulatory complications<br>E116 E11.6 With other specified complications<br>E117 E11.7 With multiple complications<br>E118 E11.8 With unspecified complications<br>E119 E11.9 Without complications<br>E12 E12 Malnutrition-related diabetes mellitus<br>E120 E12.0 With coma<br>E121 E12.1 With ketoacidosis<br>E122 E12.2 With renal complications<br>E123 E12.3 With ophthalmic complications<br>E124 E12.4 With neurological complications<br>E125 E12.5 With peripheral circulatory complications<br>E126 E12.6 With other specified complications<br>E127 E12.7 With multiple complications<br>E128 E12.8 With unspecified complications<br>E129 E12.9 Without complications<br>E13 E13 Other specified diabetes mellitus<br>E130 E13.0 With coma<br>E131 E13.1 With ketoacidosis<br>E132 E13.2 With renal complications<br>E133 E13.3 With ophthalmic complications<br>E134 E13.4 With neurological complications<br>E135 E13.5 With peripheral circulatory complications<br>E136 E13.6 With other specified complications<br>E137 E13.7 With multiple complications<br>E138 E13.8 With unspecified complications<br>E139 E13.9 Without complications<br>E14 E14 Unspecified diabetes mellitus<br>E140 E14.0 With coma<br>E141 E14.1 With ketoacidosis<br>E142 E14.2 Withrenal complications<br>E143 E14.3 With ophthalmic complications<br>E144 E14.4 With neurological complications<br>E145 E14.5 With peripheral circulatory complications<br>E146 E14.6 With other specified complications<br>E147 E14.7 With multiple complications<br>E148 E14.8 With unspecified complications<br>E149 E14.9 Without complications<br>E15 E15 Nondiabetic hypoglycaemic coma<br>**Noncancer_illness_code_selfreported_f20002_0_0** through **noncancer_illness_code_selfreported_f20002_0_33 (34 arrays)** using these codes (**interpolated_age_of_participant_when_noncancer_illness_first_diagnosed_f20009_0_0** through **interpolated_age_of_participant_when_noncancer_illness_first_diagnosed_f20009_0_33** was used to assign the age at diagnosis)::<br>1220 diabetes<br>1222 type 1 diabetes<br>1223 type 2 diabetes<br><br>(binary definition across any reports of having diabetes or taking medication for it in the phenotype data or having and the medical codes before the date of the MRI scan)
**Physical Inactivity**	**at_or_above_moderatevigorous_recommendation_f22035_2_0**<br>**at_or_above_moderatevigorouswalking_recommendation_f22036_2_0**<br>**summed_minutes_activity_f22034_2_0**<br>(at or above WHO guideline of 500 minutes per week)

**Body Mass Index**	**body_mass_index_bmi_f21001_2_0**
**Head injury**	**ICD10: diagnoses_icd10_f41270_0_0** through **diagnoses_icd10_f41270_0_258 (259 arrays)** using these ICD10 codes (**date_of_first_inpatient_diagnosis_icd10_f41280_0_0** through **date_of_first_inpatient_diagnosis_icd10_f41280_0_258** was used to define the date): :<br>S02 S02 Fracture of skull and facial bones<br>S020 S02.0 Fracture of vault of skull<br>S0200 S02.00 Fracture of vault of skull (closed)<br>S0201 S02.01 Fracture of vault of skull (open)<br>S021 S02.1 Fracture of base of skull<br>S0210 S02.10 Fracture of base of skull (closed)<br>S0211 S02.11 Fracture of base of skull (open)<br>S022 S02.2 Fracture of nasal bones<br>S0220 S02.20 Fracture of nasal bones (closed)<br>S0221 S02.21 Fracture of nasal bones (open)<br>S023 S02.3 Fracture of orbital floor<br>S0230 S02.30 Fracture of orbital floor (closed)<br>S0231 S02.31 Fracture of orbital floor (open)<br>S024 S02.4 Fracture of malar and maxillary bones<br>S0240 S02.40 Fracture of malar and maxillary bones (closed)<br>S0241 S02.41 Fracture of malar and maxillary bones (open)<br>S026 S02.6 Fracture of mandible<br>S0260 S02.60 Fracture of mandible (closed)<br>S0261 S02.61 Fracture of mandible (open)<br>S027 S02.7 Multiple fractures involving skull and facial bones<br>S0270 S02.70 Multiple fractures involving skull and facial bones (closed)<br>S0271 S02.71 Multiple fractures involving skull and facial bones (open)<br>S028 S02.8 Fractures of other skull and facial bones<br>S0280 S02.80 Fractures of other skull and facial bones (closed)<br>S0281 S02.81 Fractures of other skull and facial bones (open)<br>S029 S02.9 Fracture of skull and facial bones, part unspecified<br>S0290 S02.90 Fracture of skull and facial bones, part unspecified (closed)<br>S0291 S02.91 Fracture of skull and facial bones, part unspecified (open)<br>S06 S06 Intracranial injury<br>S060 S06.0 Concussion<br>S0600 S06.00 Concussion (without open intracranial wound)<br>S0601 S06.01 Concussion (with open intracranial wound)<br>S061 S06.1 Traumatic cerebral oedema<br>S0610 S06.10 Traumatic cerebral oedema (without open intracranial wound)<br>S0611 S06.11 Traumatic cerebral oedema (with open intracranial wound)<br>S062 S06.2 Diffuse brain injury<br>S0620 S06.20 Diffuse brain injury (without open intracranial wound)<br>S0621 S06.21 Diffuse brain injury (with open intracranial wound)<br>S063 S06.3 Focal brain injury<br>S0630 S06.30 Focal brain injury (without open intracranial wound)<br>S0631 S06.31 Focal brain injury (with open intracranial wound)<br>S064 S06.4 Epidural haemorrhage<br>S0640 S06.40 Epidural haemorrhage (without open intracranial wound)<br>S0641 S06.41 Epidural haemorrhage (with open intracranial wound)<br>S065 S06.5 Traumatic subdural haemorrhage<br>S0650 S06.50 Traumatic subdural haemorrhage (without open intracranial wound)<br>S0651 S06.51 Traumatic subdural haemorrhage (with open intracranial wound)<br>S066 S06.6 Traumatic subarachnoid haemorrhage<br>S0660 S06.60 Traumatic subarachnoid haemorrhage (without open intracranial wound)<br>S0661 S06.61 Traumatic subarachnoid haemorrhage (with open intracranial wound)<br>S067 S06.7 Intracranial injury with prolonged coma<br>S0670 S06.70 Intracranial injury with prolonged coma (without open intracranial wound)<br>S0671 S06.71 Intracranial injury with prolonged coma (with open intracranial wound)<br>S068 S06.8 Other intracranial injuries<br>S0680 S06.80 Other intracranial injuries (without open intracranial wound)<br>S0681 S06.81 Other intracranial injuries (with open intracranial wound)<br>S069 S06.9 Intracranial injury, unspecified<br>S0690 S06.90 Intracranial injury, unspecified (without open intracranial wound)<br>S0691 S06.91 Intracranial injury, unspecified (with open intracranial wound)<br>S07 S07 Crushing injury of head<br>S070 S07.0 Crushing injury of face<br>S071 S07.1 Crushing injury of skull<br>S078 S07.8 Crushing injury of other parts of head<br>S079 S07.9 Crushing injury of head, part unspecified<br>S08 S08 Traumatic amputation of part of head<br>S080 S08.0 Avulsion of scalp

	S088 S08.8 Traumatic amputation of other parts of head<br>S089 S08.9 Traumatic amputation of unspecified part of head<br>S09 S09 Other and unspecified injuries of head<br>S090 S09.0 Injury of blood vessels of head, not elsewhere classified<br>S091 S09.1 Injury of muscle and tendon of head<br>S092 S09.2 Traumatic rupture of ear drum<br>S097 S09.7 Multiple injuries of head<br>S098 S09.8 Other specified injuries of head<br>S099 S09.9 Unspecified injury of head<br>F072 F07.2 Postconcussional syndrome<br>S140 S14.0 Concussion and oedema of cervical spinal cord<br>S240 S24.0 Concussion and oedema of thoracic spinal cord<br>S340 S34.0 Concussion and oedema of lumbar spinal cord<br>T90 T90 Sequelae of injuries of head<br>T901 T90.1 Sequelae of open wound of head<br>T902 T90.2 Sequelae of fracture of skull and facial bones<br>T905 T90.5 Sequelae of intracranial injury<br>T908 T90.8 Sequelae of other specified injuries of head<br>T909 T90.9 Sequelae of unspecified injury of head<br>T903 T90.3 Sequelae of injury of cranial nerves<br>T904 T90.4 Sequelae of injury of eye and orbit<br>**Noncancer_illness_code_selfreported_f20002_0_0** through **noncancer_illness_code_selfreported_f20002_0_33 (34 arrays)** using these codes (**interpolated_age_of_participant_when_noncancer_illness_first_diagnosed_f20009_0_0** through **interpolated_age_of_participant_when_noncancer_illness_first_diagnosed_f20009_0_33** was used to assign the age at diagnosis):<br>1240 neurological injury/trauma<br>1266 head injury<br>1626 fracture skull / head
**Polygenic Risk Score for Alzheimer's Disease**	**standard_prs_for_alzheimers_disease_ad_f26206_0_0**
**Pairs Matching Test**	**number_of_incorrect_matches_in_round_f399_2_2**<br><br>(6 pairs, 1st attempt was used and data was log-transformed)
**Reaction Time Test**	**mean_time_to_correctly_identify_matches_f20023_2_0**<br><br>(data was log-transformed)
**Verbal/Numerical Reasoning Test**	**fluid_intelligence_score_f20016_2_0**
**Prospective Memory Test**	**prospective_memory_result_f20018_2_0**<br><br>(1 was assigned when the participant was correct on first attempt; otherwise, this was coded as 0)
**Volume of CA1-body (left hemisphere)**	**volume_of_ca1body_left_hemisphere_f26622_2_0**
**Volume of CA1-body (right hemisphere)**	**volume_of_ca1body_right_hemisphere_f26644_2_0**
**Volume of CA1-head (left hemisphere)**	**volume_of_ca1head_left_hemisphere_f26626_2_0**
**Volume of CA1-head (right hemisphere)**	**volume_of_ca1head_right_hemisphere_f26648_2_0**
**Volume of CA3-body (left hemisphere)**	**volume_of_ca3body_left_hemisphere_f26632_2_0**
**Volume of CA3-body (right hemisphere)**	**volume_of_ca3body_right_hemisphere_f26654_2_0**
**Volume of CA3-head (left hemisphere)**	**volume_of_ca3head_left_hemisphere_f26637_2_0**
**Volume of CA3-head (right hemisphere)**	**volume_of_ca3head_right_hemisphere_f26659_2_0**

**Volume of CA4-body (left hemisphere)**	**volume_of_ca4body_left_hemisphere_f26635_2_0**
**Volume of CA4-body (right hemisphere)**	**volume_of_ca4body_right_hemisphere_f26657_2_0**
**Volume of CA4-head (left hemisphere)**	**volume_of_ca4head_left_hemisphere_f26634_2_0**
**Volume of CA4-head (right hemisphere)**	**volume_of_ca4head_right_hemisphere_f26656_2_0**
**Volume of Hippocampal-tail (left hemisphere)**	**volume_of_hippocampaltail_left_hemisphere_f26620_2_0**
**Volume of Hippocampal-tail (right hemisphere)**	**volume_of_hippocampaltail_right_hemisphere_f26642_2_0**
**Volume of Whole-hippocampal-body (left hemisphere)**	**volume_of_wholehippocampalbody_left_hemisphere_f26639_2_0**
**Volume of Whole-hippocampal-body (right hemisphere)**	**volume_of_wholehippocampalbody_right_hemisphere_f26661_2_0**
**Volume of Whole-hippocampal-head (left hemisphere)**	**volume_of_wholehippocampalhead_left_hemisphere_f26640_2_0**
**Volume of Whole-hippocampal-head (right hemisphere)**	**volume_of_wholehippocampalhead_right_hemisphere_f26662_2_0**
**Volume of Whole-hippocampus (left hemisphere)**	**volume_of_wholehippocampus_left_hemisphere_f26641_2_0**
**Volume of Whole-hippocampus (right hemisphere)**	**volume_of_wholehippocampus_right_hemisphere_f26663_2_0**
**Volume of hippocampal-fissure (left hemisphere)**	**volume_of_hippocampalfissure_left_hemisphere_f26624_2_0**
**Volume of hippocampal-fissure (right hemisphere)**	**volume_of_hippocampalfissure_right_hemisphere_f26646_2_0**
**Volume of molecular-layer-HP-body (left hemisphere)**	**volume_of_molecularlayerhpbody_left_hemisphere_f26630_2_0**
**Volume of molecular-layer-HP-body (right hemisphere)**	**volume_of_molecularlayerhpbody_right_hemisphere_f26652_2_0**
**Volume of molecular-**	**volume_of_molecularlayerhphead_left_hemisphere_f26629_2_0**

layer-HP-head (left hemisphere)	
Volume of molecular-layer-HP-head (right hemisphere)	volume_of_molecularlayerhphead_right_hemisphere_f26651_2_0
Volume of parasubiculum (left hemisphere)	volume_of_parasubiculum_left_hemisphere_f26628_2_0
Volume of parasubiculum (right hemisphere)	volume_of_parasubiculum_right_hemisphere_f26650_2_0
Volume of presubiculum-body (left hemisphere)	volume_of_presubiculumbody_left_hemisphere_f26627_2_0
Volume of presubiculum-body (right hemisphere)	volume_of_presubiculumbody_right_hemisphere_f26649_2_0
Volume of presubiculum-head (left hemisphere)	volume_of_presubiculumhead_left_hemisphere_f26625_2_0
Volume of presubiculum-head (right hemisphere)	volume_of_presubiculumhead_right_hemisphere_f26647_2_0
Volume of subiculum-body (left hemisphere)	volume_of_subiculumbody_left_hemisphere_f26621_2_0
Volume of subiculum-body (right hemisphere)	volume_of_subiculumbody_right_hemisphere_f26643_2_0
Volume of subiculum-head (left hemisphere)	volume_of_subiculumhead_left_hemisphere_f26623_2_0
Volume of subiculum-head (right hemisphere)	volume_of_subiculumhead_right_hemisphere_f26645_2_0
Volume of grey matter in Parahippocampal Gyrus, anterior division (left)	F25848
Volume of grey matter in Parahippocampal Gyrus, anterior division (right)	F25848
Volume of grey matter in Parahippocampal Gyrus, posterior division (left)	F25850
Volume of grey matter in Parahippocampal Gyrus,	F25851

**posterior division (right)**	
