# Supplementary material for: Early detection of dementia with default-mode network effective connectivity
Source: Nat Ment Health. Author manuscript; Available in PMC 2026 Feb 18. (PMC7618740; doi:10.1038/s44220-024-00259-5)
Supplement: Supplementary Material [file EMS212461-supplement-Supplementary_Material.zip › 44220_2024_259_MOESM2_ESM.pdf]

nature portfolio

Corresponding author(s): Prof. Charles Marshall

Last updated by author(s): Apr 21, 2024

Reporting Summary

Nature Portfolio wishes to improve the reproducibility of the work that we publish. This form provides structure for consistency and transparency in reporting. For further information on Nature Portfolio policies, see our Editorial Policies and the Editorial Policy Checklist.

Statistics

For all statistical analyses, confirm that the following items are present in the figure legend, table legend, main text, or Methods section.

n/a	Confirmed	
☐	☒	The exact sample size (*n*) for each experimental group/condition, given as a discrete number and unit of measurement
☐	☒	A statement on whether measurements were taken from distinct samples or whether the same sample was measured repeatedly
☐	☒	The statistical test(s) used AND whether they are one- or two-sided<br>*Only common tests should be described solely by name; describe more complex techniques in the Methods section.*
☐	☒	A description of all covariates tested
☐	☒	A description of any assumptions or corrections, such as tests of normality and adjustment for multiple comparisons
☐	☒	A full description of the statistical parameters including central tendency (e.g. means) or other basic estimates (e.g. regression coefficient) AND variation (e.g. standard deviation) or associated estimates of uncertainty (e.g. confidence intervals)
☐	☒	For null hypothesis testing, the test statistic (e.g. *F*, *t*, *r*) with confidence intervals, effect sizes, degrees of freedom and *P* value noted<br>*Give P values as exact values whenever suitable.*
☐	☒	For Bayesian analysis, information on the choice of priors and Markov chain Monte Carlo settings
☒	☐	For hierarchical and complex designs, identification of the appropriate level for tests and full reporting of outcomes
☐	☒	Estimates of effect sizes (e.g. Cohen's *d*, Pearson's *r*), indicating how they were calculated

Our web collection on statistics for biologists contains articles on many of the points above.

Software and code

Policy information about availability of computer code

Data collection	No software was used for data collection
Data analysis	MATLAB 2023a, SPM12, DCM12, glmnet 4.1-8, custom MATLAB code available at https://github.com/Wolfson-PNU-QMUL/UKB_DCM_dementia

For manuscripts utilizing custom algorithms or software that are central to the research but not yet described in published literature, software must be made available to editors and reviewers. We strongly encourage code deposition in a community repository (e.g. GitHub). See the Nature Portfolio guidelines for submitting code & software for further information.

Data

Policy information about availability of data

All manuscripts must include a data availability statement. This statement should provide the following information, where applicable:

- Accession codes, unique identifiers, or web links for publicly available datasets
- A description of any restrictions on data availability
- For clinical datasets or third party data, please ensure that the statement adheres to our policy

Processed group-level DCM results are available at https://github.com/Wolfson-PNU-QMUL/UKB_DCM_dementia. Supplementary Table S4 contains UKB field names for UKB data variables analysed in this study.

Research involving human participants, their data, or biological material

Policy information about studies with human participants or human data. See also policy information about sex, gender (identity/presentation), and sexual orientation and race, ethnicity and racism.

Reporting on sex and gender	Participant sex identification was acquired from a central registry (NHS) at the time of recruitment to the UK Biobank, but in some cases was updated through participant self-report.
Reporting on race, ethnicity, or other socially relevant groupings	Participant ethnicity was defined through self-report at the time of recruitment to the UK Biobank. Participants were asked to report their ethnicity as "white", "mixed", "Asian or Asian British", "black or black British", "Chinese", "other ethnic group", "do not know" or "prefer not to answer".
Population characteristics	Population characteristics are provided in Extended Data Table 1
Recruitment	We identified all UKB participants who have ever had a dementia diagnosis on their health record, as of the UKB data update in May 2023, and who also had resting-state functional MRI (rs-fMRI) data available on the UKB database. Our sample size was therefore determined by data availability. By identifying every single participant with a dementia diagnosis, selection bias was mitigated in this study. This yielded an initial sample of 148 dementia cases. For each of these dementia cases, we identified 10 control participants from UKB, who did not have a dementia diagnosis on their health record, and were matched with the dementia case on age, sex, handedness, ethnicity, and geographical location of MRI scanning centre.
Ethics oversight	This research was conducted using the UKB Resource under Application Number 78867 (PI: Prof. Charles Marshall). Informed written consent was obtained from all participants on enrolment in UKB and they were informed that they are free to withdraw their consent at any time, at which time their data would be censored and excluded from future analysis. Participants were offered compensation for reasonable travel expenses. The UKB has approval from the North West Multicentre Research Ethics Committee (MREC) as a Research Tissue Bank (RTB). REC reference: 21/NW/0157.

Note that full information on the approval of the study protocol must also be provided in the manuscript.

Field-specific reporting

Please select the one below that is the best fit for your research. If you are not sure, read the appropriate sections before making your selection.

☒ Life sciences ☐ Behavioural & social sciences ☐ Ecological, evolutionary & environmental sciences

For a reference copy of the document with all sections, see nature.com/documents/nr-reporting-summary-flat.pdf

Life sciences study design

All studies must disclose on these points even when the disclosure is negative.

Sample size	N = 1133. We identified all UKB participants who have ever had a dementia diagnosis on their health record, as of the UKB data update in May 2023, and who also had resting-state functional MRI (rs-fMRI) data available on the UKB database. Our sample size was therefore determined by data availability.
Data exclusions	After excluding participants who failed the preprocessing stage (e.g. excessive head motion) and replacing failed controls with new matched controls we were left with a final usable sample of 103 cases and 1030 matched controls. Of these 103 cases, 81 did not have a dementia diagnosis at the time of MRI data acquisition, whilst 22 already had prevalent dementia. In total, 1485 control participants were screened through data preprocessing before the target number of 1030 was achieved.
Replication	Replication was not performed as part of this study due to limitations in data availability. This is acknowledged in the limitations section of the Discussion in the main manuscript.
Randomization	This was a case-control study. Randomisation was not performed.
Blinding	Blinding of investigators was not feasible in this study as it was a case-control study with 10-fold more controls than cases.

Reporting for specific materials, systems and methods

We require information from authors about some types of materials, experimental systems and methods used in many studies. Here, indicate whether each material, system or method listed is relevant to your study. If you are not sure if a list item applies to your research, read the appropriate section before selecting a response.

Materials & experimental systems

n/a	Involved in the study
☒	☐ Antibodies
☒	☐ Eukaryotic cell lines
☒	☐ Palaeontology and archaeology
☒	☐ Animals and other organisms
☐	☒ Clinical data
☒	☐ Dual use research of concern
☒	☐ Plants

Methods

n/a	Involved in the study
☒	☐ ChIP-seq
☒	☐ Flow cytometry
☐	☒ MRI-based neuroimaging

Clinical data

Policy information about clinical studies

All manuscripts should comply with the ICMJE guidelines for publication of clinical research and a completed CONSORT checklist must be included with all submissions.

Clinical trial registration	No new clinical data was collected as part of this study and this was not a registered clinical trial.
Study protocol	This was a nested case-control study. The full protocol can be found in the Methods section of the manuscript.
Data collection	No data collection was carried out as part of this study. Pre-existing data from the UK Biobank was downloaded and analysed.
Outcomes	Outcomes were a) Dementia incidence, b) Time until dementia diagnosis. These were established using NHS records.

Plants

Seed stocks	*Report on the source of all seed stocks or other plant material used. If applicable, state the seed stock centre and catalogue number. If plant specimens were collected from the field, describe the collection location, date and sampling procedures.*
Novel plant genotypes	*Describe the methods by which all novel plant genotypes were produced. This includes those generated by transgenic approaches, gene editing, chemical/radiation-based mutagenesis and hybridization. For transgenic lines, describe the transformation method, the number of independent lines analyzed and the generation upon which experiments were performed. For gene-edited lines, describe the editor used, the endogenous sequence targeted for editing, the targeting guide RNA sequence (if applicable) and how the editor was applied.*
Authentication	*Describe any authentication procedures for each seed stock used or novel genotype generated. Describe any experiments used to assess the effect of a mutation and, where applicable, how potential secondary effects (e.g. second site T-DNA insertions, mosiacism, off-target gene editing) were examined.*

Magnetic resonance imaging

Experimental design

Design type	Case-control analysis - comparing effective connectivity maps between two groups
Design specifications	N = 103 cases, N = 1030 controls. Effective connectivity estimated using DCM.
Behavioral performance measures	None (resting-state fMRI)

Acquisition

Imaging type(s)	Resting-state fMRI
Field strength	3T
Sequence & imaging parameters	Resolution 2.4 mm isotropic, TR 0.735 s, TE 39 ms, flip angle 52
Area of acquisition	Whole brain

Diabetes MRI ☐ Used ☒ Not used

Preprocessing

Preprocessing software	SPM12

Normalization	Normalized to MNI space
Normalization template	MNI
Noise and artifact removal	Participants with excessive head motion (>2.4 mm framewise displacement) excluded from analysis
Volume censoring	Nil

Statistical modeling & inference

Model type and settings	Dynamic causal modelling
Effect(s) tested	Effective connectivity parameter differences between cases and controls

Specify type of analysis: ☐ Whole brain ☒ ROI-based ☐ Both

Anatomical location(s): Literature-based ROIs to define a 10-node default-mode network (DMN)

Statistic type for inference (See Eklund et al. 2016)	Parametric empirical Bayes
Correction	Nil

Models & analysis

n/a	Involved in the study
☐	☒ Functional and/or effective connectivity
☒	☐ Graph analysis
☐	☒ Multivariate modeling or predictive analysis

Functional and/or effective connectivity	Effective connectivity parameter estimates (and comparative Fisher Z-transformed Pearson coefficients also calculated)
Multivariate modeling and predictive analysis	Stratified nested K-fold cross-validation using elastic net regularised linear and logistic regression models
